# Observational analysis of factors associated with completion of four or more antenatal care visits in Sarlahi district, Nepal

**DOI:** 10.1136/bmjopen-2024-098478

**Published:** 2026-03-10

**Authors:** Yiwei Yue, Elizabeth A Hazel, Seema Subedi, Scott Zeger, Diwakar Mohan, Luke C Mullany, James M Tielsch, Subarna Khatry, Steven C LeClerq, Joanne Katz

**Affiliations:** 1 Department of International Health, Johns Hopkins Bloomberg School of Public Health, Baltimore, Maryland, USA; 2 Department of Mental Health, Johns Hopkins Bloomberg School of Public Health, Baltimore, MD, USA; 3 Department of Biostatistics, Johns Hopkins Bloomberg School of Public Health, Baltimore, MD, USA; 4 Department of Global Health, Milken Institute School of Public Health, George Washington University, Washington, DC, USA; 5 Nepal Nutrition Intervention Project, Lalitpur, Nepal

**Keywords:** Maternal health, maternal and newborn healthcare, pregnancy, antenatal care

## Abstract

**Background:**

A significant number of women die from pregnancy and childbirth complications globally, particularly in low and middle-income countries. Receiving at least four antenatal care (ANC) visits is important in reducing maternal and perinatal deaths. However, few studies have investigated the factors linked to the completion of ≥4 ANC visits in Nepal.

**Objective:**

To investigate factors associated with attending ≥4 ANC visits in Sarlahi district of southern Nepal.

**Design, setting and participants:**

A secondary analysis was conducted on data from the Nepal Oil Massage Study (NOMS), a cluster-randomised, community-based longitudinal pregnancy trial including 34 village development committees. We investigated the associations between attendance of ≥4 ANC visits and socioeconomic, demographic, morbidity and pregnancy history factors using logistic regression; generalised estimating equations were used to account for multiple pregnancies per woman. All pregnancies resulting in a live birth (LB) (n=31 867) were included.

**Primary and secondary outcome measures:**

Attendance of ≥4 ANC visits.

**Results:**

31.4% of those pregnancies received 4+ ANC visits. Significant positive associations included socioeconomic factors such as participation in non-farming occupations for women (OR=1.52, 95% CI 1.19 to 1.93), higher education (OR=1.79, 95% CI 1.66 to 1.93) and wealth quintile (OR=1.44, 95% CI 1.31 to 1.59), nutritional status such as non-short stature (OR=1.17, 95% CI 1.07 to 1.27), obstetric history such as adequate interpregnancy interval (OR=1.31, 95% CI 1.19 to 1.45) and prior pregnancy but no LB (OR=2.14, 95% CI 1.57 to 2.92), symptoms such as vaginal bleeding (OR=1.35, 95% CI 1.11 to 1.65) and awareness of the government’s conditional cash transfer ANC programme (OR=2.26, 95% CI 2.01 to 2.54). Conversely, identifying as the Shudra caste (OR=0.56, 95% CI 0.47 to 0.67), maternal age below 18 or above 35 (OR=0.81, 95% CI 0.74 to 0.88; OR=0.77, 95% CI 0.62 to 0.96), preterm birth (OR=0.41, 95% CI 0.35 to 0.49), parity ≥1 (OR=0.66, 95% CI 0.61 to 0.72) and the presence of hypertension during pregnancy (OR=0.79, 95% CI 0.69 to 0.90) were associated with decreased likelihood of attending ≥4 ANC visits.

**Conclusions:**

These findings demonstrate the importance of socioeconomic factors, including education, caste, wealth and occupation in completion of ≥4 ANC visits. In addition, biological factors including birth spacing, pregnancy complications and nutrition are important. The association with awareness of the government’s conditional cash transfer programme is a motivation for a full evaluation of whether expanding that programme might improve prenatal care.

**Trial registration number:**

The clinicaltrial.gov trial registration number for NOMS was NCT01177111.

**STRENGTHS AND LIMITATIONS OF THIS STUDY**

- Used a population-based pregnancy trial with large sample size, enabling prospective measurements of potential risk factors throughout pregnancies.
- Investigated the prospective associations between multilevel factors (ie, socioeconomic and demographic factors, reproductive history related factors, morbidity during pregnancy) and the receipt of ≥4, ≥3, ≥5 and ≥8 antenatal care (ANC) visits in Nepal.
- Challenges in establishing causal relationships due to the observational study design and potential confounders not accounted for. Maternal recall of ANC visits may introduce recall bias, with validity potentially differing.
- Generalisability may be limited, particularly in urban settings where factors linked to receipt of ≥4 ANC visits may differ.
- Self-reported data may introduce response biases, and the absence of first-trimester morbidity data and ANC quality evaluation may impact findings.

## Background

 Despite improvements in global maternal health, the number of women dying due to pregnancy and childbirth complications in 2020 remains high, with estimates of 800 maternal deaths daily.^1^ Only 69% of pregnant women completed at least four antenatal care (ANC) visits globally in 2022.^1^ Furthermore, around 95% of preventable maternal deaths occurred in low and middle-income countries (LMICs) in 2020, highlighting the disparities and inequalities in health services.^1^

The 2016 Nepal Demographic and Health Survey (NDHS) reported a high pregnancy-related mortality ratio (259 maternal deaths per 100 000 live births (LBs)) and a high maternal mortality ratio (MMR) (239 maternal deaths per 100 000 LBs) during the 7 years preceding the survey.^2^ The levels of maternal mortality in Nepal have improved since 2016. Based on the Nepal Maternal Mortality Study 2021, the latest MMR was 151 maternal deaths per 100 000 LBs in Nepal.^3^

In addition, infant and child health improvement remained slow in Nepal between 2016 and 2022.^2 4^ The 2022 NDHS indicated that the neonatal mortality rate (NMR) remained the same as that of the 2016 NDHS (ie, NMR: 21 deaths per 1000 LBs).^2 4^ The infant mortality rate fell from 32 to 28 deaths per 1000 LBs and the under-5 mortality rate (from 39 to 33 deaths per 1000 LBs) declined between 2016 and 2022.^2 4^

According to the 2016 NDHS, ANC attendance and maternal health service utilisation in Nepal were below the global average, with 84% of women attending any ANC visit with skilled providers, 69% with 4+ ANC visits, 57% delivered in a health facility, 58% with delivery assisted by a skilled provider and 57% postnatal care coverage (mothers and newborns received a postnatal check-up within 2 days of delivery by a skilled provider) in the 5 years before the survey.^2^ The 2022 NDHS showed improvements in maternal and newborn healthcare since 2016.^2 4^ In the 2 years prior to the 2022 survey, 94% of women had ANC from a skilled provider for their most recent LB or stillbirth (SB).^4^ Additionally, 80% of women attended ≥4 ANC visits, 79% of deliveries occurred in a health facility, 80% of deliveries were conducted by skilled providers and 70% of women and newborns received a postnatal check.^4^

Receiving an adequate number of ANC visits is associated with improved maternal and newborn health.^5^ Specifically, a large community-based cluster-randomised trial, the Nepal Oil Massage Study (NOMS), examined the impact of switching from mustard to sunflower seed oil for newborn massage on all-cause mortality, mortality among preterm babies and signs of infection in Nepal from 2010 to 2017.^6^ Additionally, the NOMS study found that there was an estimated 33% lower neonatal mortality among babies born to women attending at least four ANC sessions.^5^ During most of the NOMS study period, the Nepal government and the WHO recommended at least four ANC visits during pregnancy from 2002 until 2016, and then WHO updated the recommendation to eight ANC visits in 2016,^7^,^9^ but this was not policy in Nepal during the time period of the study.

Regular and quality ANC during pregnancy are important to detect and intervene in pregnancy risks, improve the health outcomes of pregnant women and newborns, and reduce maternal and neonatal mortality and morbidity.^10^,^13^ An adequate number of ANC visits and quality ANC services mitigate risks including anaemia, pregnancy-induced hypertension, preterm labour, preterm births and low birth weight.^1214^,^16^ ANC visits serve not only as a preventative measure but also provide education to women regarding childbirth, family planning and birth spacing.^17^ Additionally, ANC encourages institutional deliveries, reduces maternal mortality and improves neonatal outcomes.^17^

Multiple factors have been linked to ANC utilisation in Nepal, such as socioeconomic and demographic factors (eg, education, wealth, caste, occupation, ethnicity, age, and cash incentives) to encourage the completion of ≥4 visits.^18 19^ Although there have been national reports on ANC coverage and its components,^2 4^ few studies have examined the prospective associations between multi-level factors and the completion of ≥4 ANC visits among a large population in Nepal. ANC coverage remains uneven across Nepal, and Sarlahi district is considered a poor region of Nepal.^2 4 6^ Therefore, it is important to identify district-specific factors associated with completing ≥4 ANC visits, particularly for marginalised populations such as pregnant women in rural Sarlahi District, Nepal. Additionally, less is known about the associations between pregnancy history and prior pregnancy outcomes with the attendance of ANC visits in Nepal. Therefore, we aimed to assess the association between demographic factors, socioeconomic status (SES), pregnancy history, morbidity symptoms in pregnancy and the frequency of ANC visits by women in Sarlahi district of Nepal using NOMS data. These findings will inform interventions to increase ANC visits and improve maternal and neonatal health in Nepal and possibly similar LMICs.

## Methods

### Study design

This study is an observational secondary analysis of data collected during the NOMS. The NOMS was a cluster-randomised community-based controlled trial that included mothers and their live and stillborn infants in 34 village development committees in Sarlahi District, rural southern Nepal, from 2010 to 2017.^6^ A total of 340 geographic clusters were randomised to receive either sunflower seed oil to massage for newborn babies or the standard mustard seed oil.^6^ Specifically, the NOMS treatment (ie, either sunflower seed oil or the standard mustard seed oil for newborn babies’ massages) was only applied after the birth of the baby, rather than during pregnancy.^6^ Therefore, the treatment and accounting for randomisation to clusters is not relevant to the outcome of the number of ANC visits attended.^6^ Additional details about the NOMS study are available elsewhere, including the main NOMS publication.^6^ The NOMS study manual of operation is available in online supplemental file 1.

We cannot say for sure that missing data are missing at random. However, there do not appear to be differences in characteristics available to us between missing and available data, implying missing at random is likely.

### Participants

The population for analysis consisted of married women of reproductive age for incident pregnancies that resulted in at least one LB within NOMS.^6^ Since the trial enrolled and followed women over several years, some women contributed more than one pregnancy to the analysis. We excluded pregnancies with missing values or SBs or abortions, only including LBs for analysis. We did not include pregnancies resulting in fetal loss because the number of ANC visits was not collected on them. All pregnancies that resulted in at least one LB were included, regardless of their intervention assignment, as intervention for the NOMS trial occurred after delivery, and our ANC outcome (ie, completion of ≥4 ANC visits) occurred before delivery.

Married women of childbearing age were visited by locally residing female workers for pregnancy surveillance visits every 5 weeks and enrolled with consent when pregnant.^6^ During monthly intervals from enrolment to pregnancy outcome, field workers conducted home visits to confirm pregnancy status, document morbidity symptoms in the prior 30 days, and measure weight, blood pressure, pulse and body temperature.^6^

### Measures

The number of ANC visits women attended during pregnancy was obtained from maternal recall by study staff during home visits as soon as possible after delivery. Our primary ANC visit outcome was binarised as four or more ANC visits, and <4 ANC visits (including no visits).

Predictors including demographic, SES, and obstetric and medical history were collected at enrolment. Pregnancy information such as morbidity symptoms was collected monthly throughout pregnancy, including morbidity recall and measurement of pulse, temperature and blood pressure. As soon as possible after birth, study staff interviewed the mother about labour and delivery and recorded the pregnancy outcome (eg, LBs, SBs, multiple births). Awareness of a government cash transfer programme that incentivised women to receive ANC and deliver at a facility was noted through maternal recall. We did not adjust for smoking or alcohol because the use of smoking or alcohol was low (tobacco: 1.1%, alcohol 0.3%) in the NOMS trial.^5^

Women’s and their husbands’ occupations were grouped into four categories: (1) Farmer, (2) Does not work outside home, (3) Unskilled or day or contracted labourer and (4) Business or private or government service. Maternal education was divided into three levels: none, 1–5 years and over 5 years of formal education. Wealth was classified into five quintiles based on assets and living conditions.^12 20^ Caste/religion was categorised into four groups: Brahmin and Chhetri, Vaishya, Shudra and Muslim and others, with Brahmin and Chhetri being the two highest castes in the study setting. Maternal age groups were: <18 years, 18–35 and >35 years of age at enrolment. Maternal height was divided into three ranges: <145 centimeters (cm), 145–150 cm and ≥150 cm. Gestational age was classified as moderately preterm (<34 weeks), mildly preterm (34–37 weeks) and term (≥37 weeks). Parity was categorised into three groups: no prior pregnancy, prior pregnancy but no live or SB and parity ≥1. Pregnancy history/outcome along with survival of any babies born was divided into four groups: prior LB survived, prior LB died, prior pregnancy but no LB and no prior pregnancy. Prior pregnancy history concerning SB was divided into three categories: prior pregnancy but no SB, prior SB and no prior pregnancy. Interpregnancy interval (IPI) was divided into four groups: 18 to 36 months, <18 months, >36 months and no prior pregnancy. Maternal morbidities like fever, tachycardia, hypertension, respiratory illnesses, swelling of hands and face, gastrointestinal symptoms, vaginal bleeding and sexually transmitted illness (STI) were binary variables (present at any time during second or third trimesters). Detailed explanations for predictors are shown in online supplemental table 1.

### Statistical analyses

To examine factors associated with the completion of ≥4 ANC visits, we fit generalised estimating equation (GEE) models with a logit link and an exchangeable working correlation structure, to account for the within-person correlation arising from including more than one pregnancy for many women in the study. The predictors included women’s occupation, husband’s occupation, maternal age, maternal height, caste/ethnicity, women’s education, wealth quintile, gestational age at birth, multiple births, parity, IPI, prior LBs that died, prior pregnancies that ended in SB, conditional cash transfer programme knowledge, year of childbirth, morbidity symptoms in pregnancy at any time in either the second and/or third trimesters, including fever, tachycardia, hypertension, respiratory problems, poor appetite, nausea and vomiting, vaginal bleeding, swelling of hands and face and STI. The binary outcome is the receipt of ≥4 ANC visits. Each predictor was tested within a univariable logistic regression model via GEE. In the multivariable GEE logistic regression model, all the predictors were included in the model. We presented both the crude and adjusted OR of ≥4 ANC visits with the corresponding 95% CIs.

At the time of this study, Nepal’s policy was to promote 4 ANC visits in pregnancy, but WHO is now recommending eight or more contacts during pregnancy.^7 21^ With changing recommendations, it would be important to examine factors that are associated with ANC attendance at the new recommendation of 8 visits, as well as other cut-offs of ANC visits. Therefore, we also conducted sensitivity analysis to test the associations of longitudinal risk factors with the completion of ≥3, ≥5 and ≥8 ANC visits. All analyses were conducted using Stata software version SP V.17.0 (Stata 2021).

## Results

The distributions of population characteristics are shown in table 1, overall, and by number of ANC visits <4 and ≥4. A total of 31 867 pregnancies with LB outcomes from 24 428 women were included in our analyses. Around a tenth (11.0%, n=3517) of pregnancies were missing the reported number of ANC visits. In our study population, accounting for pregnancies with missing number of ANC visits, there were 28.0% with ≥4 ANC visits and 61.0% pregnancies with <4 ANC visits, with the average number of ANC visits in the whole analyses sample being 2.4±1.8. Among 28 350 pregnancies with non-missing numbers of ANC visits, there were 31.4% with ≥4 ANC visits and 68.6% of pregnancies with <4 ANC visits. Specifically, out of 19 443 pregnancies with fewer than four ANC visits, 28.4% had no ANC visits, 21.3% had one ANC visit, 26.1% had two ANC visits, and 24.2% had three ANC visits.

**Table 1:** Characteristics of the whole analysis sample and stratified by ≥4 antenatal care visits or not

	Total	0–3 ANC	≥4 ANC
	n=28 350	n=19 443	n=8907
Women occupation			
Farmer	1558	1162 (74.58%)	396 (25.42%)
Does not work outside home	25 639	17 492 (68.22%)	8147 (31.78%)
Unskilled labourer/day labour or contracted labourer	730	588 (80.55%)	142 (19.45%)
Business or private or government service	395	181 (45.82%)	214 (54.18%)
Missing	28	20 (71.43%)	8 (28.57%)
Husband occupation			
Farmer	7745	5465 (70.56%)	2280 (29.44%)
Does not work outside home	729	450 (61.73%)	279 (38.27%)
Unskilled labourer/day labour or contracted labourer	13 261	9550 (72.02%)	3711 (27.98%)
Business or private or government service	6580	3957 (60.14%)	2623 (39.86%)
Missing	35	21 (60.00%)	14 (40.00%)
Maternal age			
18–35	23 365	15 954 (68.28%)	7411 (31.72%)
<18	4315	2966 (68.74%)	1349 (31.26%)
More than 35	668	521 (77.99%)	147 (22.01%)
Missing	2	2 (100.00%)	(0.00%)
Maternal height (centimetre)			
<145	4186	3096 (73.96%)	1090 (26.04%)
145–150	8509	5915 (69.51%)	2594 (30.49%)
≥150	15 609	10 397 (66.61%)	5212 (33.39%)
Missing	46	35 (76.09%)	11 (23.91%)
Caste/religion			
Brahmin and Chhetri	863	449 (52.03%)	414 (47.97%)
Vaishya	20 429	13 809 (67.60%)	6620 (32.40%)
Shudra	4431	3368 (76.01%)	1063 (23.99%)
Muslim and other religions	2599	1795 (69.07%)	804 (30.93%)
Missing	28	22 (78.57%)	6 (21.43%)
Mother’s education			
No schooling	19 360	14 434 (74.56%)	4926 (25.44%)
1–5 years	2392	1520 (63.55%)	872 (36.45%)
More than 5 years	6569	3468 (52.79%)	3101 (47.21%)
Missing	29	21 (72.41%)	8 (27.59%)
Wealth quintile			
Poorest	5875	4660 (79.32%)	1215 (20.68%)
Poorer	5753	4171 (72.50%)	1582 (27.50%)
Middle	5640	3825 (67.82%)	1815 (32.18%)
Richer	5545	3504 (63.19%)	2041 (36.81%)
Richest	5518	3269 (59.24%)	2249 (40.76%)
Missing	19	14 (73.68%)	5 (26.32%)
Gestational age (weeks)			
Term: ≥37 weeks	24 119	16 099 (66.75%)	8020 (33.25%)
Mildly preterm: 34–37 weeks	3059	2347 (76.72%)	712 (23.28%)
Moderately preterm: <34 weeks	1169	994 (85.03%)	175 (14.97%)
Missing	3	3 (100.00%)	(0.00%)
Multiple births			
Singleton	28 120	19 274 (68.54%)	8846 (31.46%)
Twin/triplet	230	169 (73.48%)	61 (26.52%)
Parity			
No prior pregnancy	7917	4907 (61.98%)	3010 (38.02%)
Prior pregnancy but no live or stillbirth	687	361 (52.55%)	326 (47.45%)
Parity ≥1	19 591	14 062 (71.78%)	5529 (28.22%)
Missing	155	113 (72.90%)	42 (27.10%)
Interpregnancy interval			
18–36 months	7204	5211 (72.33%)	1993 (27.67%)
<18 months	10 274	7402 (72.05%)	2872 (27.95%)
More than 36 months	2942	1911 (64.96%)	1031 (35.04%)
No prior pregnancy	7917	4907 (61.98%)	3010 (38.02%)
Missing	13	12 (92.31%)	1 (7.69%)
Any prior live birth (LB) died			
Prior LB survived	15 772	11 276 (71.49%)	4496 (28.51%)
Prior LB died	3291	2442 (74.20%)	849 (25.80%)
Prior pregnancy but no LB	929	490 (52.74%)	439 (47.26%)
No prior pregnancy	7917	4907 (61.98%)	3010 (38.02%)
Missing	441	328 (74.38%)	113 (25.62%)
Any prior pregnancy ended in stillbirth (SB)			
Prior pregnancy but no SB	19 178	13 681 (71.34%)	5497 (28.66%)
Prior SB	1239	846 (68.28%)	393 (31.72%)
No prior pregnancy	7917	4907 (61.98%)	3010 (38.02%)
Missing	16	9 (56.25%)	7 (43.75%)
Knowledge of conditional cash transfer programme			
No	2450	2131 (86.98%)	319 (13.02%)
Yes	25 565	17 013 (66.55%)	8552 (33.45%)
Missing	335	299 (89.25%)	36 (10.75%)
Year of childbirth			
2010	378	314 (83.07%)	64 (16.93%)
2011	2821	2311 (81.92%)	510 (18.08%)
2012	3013	2299 (76.30%)	714 (23.70%)
2013	2892	2316 (80.08%)	576 (19.92%)
2014	5820	3984 (68.45%)	1836 (31.55%)
2015	5713	3385 (59.25%)	2328 (40.75%)
2016	7270	4581 (63.01%)	2689 (36.99%)
2017	441	251 (56.92%)	190 (43.08%)
Missing	2	2 (100.00%)	(0.00%)
Fever			
No	25 256	17 288 (68.45%)	7968 (31.55%)
Yes	2987	2075 (69.47%)	912 (30.53%)
Missing	107	80 (74.77%)	27 (25.23%)
Tachycardia			
No	12 508	8584 (68.63%)	3924 (31.37%)
Yes	15 757	10 795 (68.51%)	4962 (31.49%)
Missing	85	64 (75.29%)	21 (24.71%)
Hypertension			
No	26 996	18 447 (68.33%)	8549 (31.67%)
Yes	1274	936 (73.47%)	338 (26.53%)
Missing	80	60 (75.00%)	20 (25.00%)
Poor appetite, nausea and vomiting			
No	14 559	10 397 (71.41%)	4162 (28.59%)
Yes	13 709	8984 (65.53%)	4725 (34.47%)
Missing	82	62 (75.61%)	20 (24.39%)
Respiratory problem			
No	17 853	12 486 (69.94%)	5367 (30.06%)
Yes	10 415	6895 (66.20%)	3520 (33.80%)
Missing	82	62 (75.61%)	20 (24.39%)
Swelling (hands or face)			
No	26 790	18 440 (68.83%)	8350 (31.17%)
Yes	1479	942 (63.69%)	537 (36.31%)
Missing	81	61 (75.31%)	20 (24.69%)
Sexually transmitted illness			
No	22 293	15 476 (69.42%)	6817 (30.58%)
Yes	5975	3905 (65.36%)	2070 (34.64%)
Missing	82	62 (75.61%)	20 (24.39%)
Vaginal bleeding			
No	27 775	19 080 (68.69%)	8695 (31.31%)
Yes	493	301 (61.05%)	192 (38.95%)
Missing	82	62 (75.61%)	20 (24.39%)

Note: LB, live birth; SB, stillbirth.

* Morbidity symptoms in pregnancy are present at any time in either the second and/or third trimester.

ANC, antenatal care.

Results of the main GEE regression model (ie, crude and adjusted OR, 95% CI) investigating the associations between factors and the receipt of ≥4 ANC visits are summarised in table 2. In sensitivity analysis, the results remained similar, as shown in online supplemental tables 2–4.

**Table 2:** Longitudinal risk factors for ≥4 antenatal care visits

	Crude OR	95% CI	Adjusted OR	95% CI
Women occupation				
Farmer	Reference	Reference	Reference	Reference
Does not work	1.31**	**1.17 to 1.46**	0.91	0.81 to 1.04
Unskilled/day labour/contracted labourer	0.76**	**0.62 to 0.92**	0.94	0.75 to 1.18
Business/private/government service	3.16**	**2.52 to 3.95**	1.52**	1.19 to 1.93
Husband occupation				
Farmer	Reference	Reference	Reference	Reference
Does not work	1.43**	**1.22 to 1.66**	1.14	0.96 to 1.35
Unskilled labourer/day labour or contracted labourer	0.94*	**0.88 to 1.00**	1.10*	**1.02 to 1.18**
Business/private service/government service	1.54**	**1.44 to 1.65**	1.28**	**1.19 to 1.39**
Maternal age (years)				
18–35	Reference	Reference	Reference	Reference
<18	0.96	0.90 to 1.03	0.81**	**0.74 to 0.88**
>35	0.61**	**0.50 to 0.73**	0.77*	**0.62 to 0.96**
Maternal height (cm)				
<145	Reference	Reference	Reference	Reference
145–150	1.25**	**1.15 to 1.36**	1.15**	**1.05 to 1.26**
≥150	1.42**	**1.31 to 1.54**	1.17**	**1.07 to 1.27**
Caste/ethnicity				
Brahmin and Chhetri	Reference	Reference	Reference	Reference
Vaishya	0.52**	**0.46 to 0.60**	0.66**	**0.57 to 0.77**
Shudra	0.35**	**0.30 to 0.40**	0.56**	**0.47 to 0.67**
Muslim and others	0.48**	**0.41 to 0.56**	0.67**	**0.56 to 0.80**
Women education (years)				
No schooling	Reference	Reference	Reference	Reference
1–5	1.64**	**1.50 to 1.80**	1.38**	**1.26 to 1.53**
>5	2.61**	**2.46 to 2.78**	1.79**	**1.66 to 1.93**
Wealth quintile				
Poorest	Reference	Reference	Reference	Reference
Poorer	1.41**	**1.30 to 1.53**	1.19**	**1.09 to 1.31**
Middle	1.77**	**1.63 to 1.92**	1.39**	**1.26 to 1.52**
Richer	2.14**	**1.97 to 2.33**	1.44**	**1.31 to 1.59**
Richest	2.56**	**2.35 to 2.78**	1.40**	**1.26 to 1.55**
Gestational age (weeks)				
Term (≥37)	Reference	Reference	Reference	Reference
Mildly preterm (34–37)	0.63**	**0.58 to 0.68**	0.67**	**0.61 to 0.73**
Moderately preterm (<34)	0.38**	**0.32 to 0.44**	0.41**	**0.35 to 0.49**
Multiple birth				
Singleton	Reference	Reference	Reference	Reference
Twin/triplet	0.80	0.60 to 1.07	1.20	0.88 to 1.64
Parity				
No prior pregnancy	Reference	Reference	Reference	Reference
Prior pregnancy but no live or stillbirth	1.47**	**1.26 to 1.72**	0.73	0.52 to 1.04
Parity ≥1	0.68**	**0.65 to 0.72**	0.66**	**0.61 to 0.72**
Interpregnancy interval (months)				
18–36	Reference	Reference	Reference	Reference
<18	1.01	0.94 to 1.07	0.96	0.89 to 1.03
>36	1.37**	**1.25 to 1.49**	1.31**	**1.19 to 1.45**
No prior pregnancy	1.49**	**1.40 to 1.60**	–	–
Prior live birth (LB) died				
Prior LB survived	Reference	Reference	Reference	Reference
Prior LB died	0.90*	**0.82 to 0.97**	1.05	0.96 to 1.15
Prior pregnancy but no LB	2.12**	**1.86 to 2.42**	2.14**	**1.57 to 2.92**
No prior pregnancy	1.45**	**1.37 to 1.53**	–	–
Prior pregnancy ended in stillbirth (SB)				
Prior pregnancy but no SB	Reference	Reference	Reference	Reference
Prior SB	1.14*	**1.01 to 1.29**	1.12	0.95 to 1.32
No prior pregnancy	1.43**	**1.36 to 1.51**	–	–
Conditional cash transfer programme knowledge	2.96**	**2.66 to 3.30**	2.26**	**2.01 to 2.54**
Year of childbirth	1.22**	**1.20 to 1.23**	1.19**	**1.17 to 1.22**
Fever				
No	Reference	Reference	Reference	Reference
Yes	0.98	0.90 to 1.06	1.05	0.96 to 1.15
Tachycardia				
No	Reference	Reference	Reference	Reference
Yes	1.01	0.96 to 1.06	0.99	0.93 to 1.04
Hypertension				
No	Reference	Reference	Reference	Reference
Yes	0.78**	**0.69 to 0.88**	0.79**	**0.69 to 0.90**
Respiratory problem				
No	Reference	Reference	Reference	Reference
Yes	1.18**	**1.12 to 1.24**	1.12**	**1.06 to 1.19**
Poor appetite, nausea, and vomiting				
No	Reference	Reference	Reference	Reference
Yes	1.29**	**1.23 to 1.36**	1.17**	**1.11 to 1.24**
Vaginal bleeding				
No	Reference	Reference	Reference	Reference
Yes	1.34**	**1.12 to 1.61**	1.35**	**1.11 to 1.65**
Swelling of hands and face				
No	Reference	Reference	Reference	Reference
Yes	1.25**	**1.13 to 1.40**	1.26**	**1.12 to 1.41**
Sexually transmitted illness				
No	Reference	Reference	Reference	Reference
Yes	1.19**	**1.12 to 1.27**	1.24**	**1.16 to 1.33**

Note: **p<0.01 to *p<0.05.

* Morbidity symptoms in pregnancy are present at any time in either the second and/or third trimester.

### Results: socioeconomic and demographic factors

Women engaged in non-agricultural professions, such as business, private service or government service, were more likely to have ≥4 ANC visits than women who were farmers (adjusted OR=1.52, 95% CI (1.19 to 1.93)). Women whose husbands worked in business, private or government service were more likely to attend ≥4 ANC visits compared with those whose husbands were farmers (adjusted OR=1.28, 95% CI (1.19 to 1.39)). Moreover, women with husbands working as unskilled labourers, day labourers or contracted labourers were more likely to have ≥4 ANC visits when compared with the farmer husband group (adjusted OR=1.10, 95% CI (1.02 to 1.18)).

Women between 18 and 35 years of age had the highest probability of attending ≥4 ANC sessions. In comparison to this age group, women younger than 18 years or those older than 35 years were less likely to attend 4+ ANC visits (adjusted OR=0.81, 95% CI (0.74 to 0.88)) and (adjusted OR=0.77, 95% CI (0.62 to 0.96)), respectively. Taller women had a higher probability of attending ≥4 ANC sessions (adjusted OR=1.15, 95% CI (1.05 to 1.26)) for those between 145 cm and 150 cm, and those ≥150 cm (adjusted OR=1.17, 95% CI (1.07 to 1.27)) when compared with women shorter than 145 cm. Compared with the upper castes of Brahmin and Chhetri, Vaishya, Shudra and Muslim and others, women had 34%, 44% and 33% lower odds of attending ≥4 ANC sessions.

Women with one to five years of education were more likely to have ≥4 ANC sessions than those without any education (adjusted OR=1.38, 95% CI (1.26 to 1.53)). Those with over 5 years of education were more likely to attend ≥4 ANC visits compared with their non-educated counterparts (adjusted OR=1.79, 95% CI (1.66 to 1.93)). While there was no consistent increasing trend across wealth quintiles, women from the wealthiest quintile were more likely to attend ≥4 ANC visits compared with those from the poorest quintile (adjusted OR=1.40, 95% CI (1.26 to 1.55)).

From 2010 to 2017, women had an increased probability of attending ≥4 ANC visits per year (adjusted OR=1.19, 95% CI (1.17 to 1.22)). The prevalence of ≥4 ANC visits by child’s birth year increased across time as follows: 64 (16.9%) in 2010, 510 (18.1%) in 2011, 714 (23.7%) in 2012, 576 (19.9%) in 2013, 1836 (31.6%) in 2014, 2328 (40.8%) in 2015, 2689 (37.0%) in 2016 and 190 (43.1%) in 2017, not including missing values in the denominator.

Additionally, women who were aware of the government’s conditional cash transfer programme promoting ≥4 ANC visits and facility-based delivery were more likely to attend ≥4 ANC visits compared with those who were unaware of the programme (adjusted OR=2.26, 95% CI (2.01 to 2.54)). The prevalence of awareness of the government’s conditional cash transfer programme promoting ≥4 ANC visits increased over time: 254 (66.8%) in 2010, 2286 (82.1%) in 2011, 2472 (84.3%) in 2012, 2470 (88.1%) in 2013, 5408 (93.7%) in 2014, 5400 (94.7%) in 2015, 6891 (95.1%) in 2016 and 428 (96.8%) in 2017, not including missing values in the denominator.

We conducted a formal trend analysis to investigate trends across the child’s birth years for both awareness of the government’s conditional cash transfer programme and 4+ ANC visits. There were increasing trends across the study period (2010–2017) for both awareness of the conditional cash transfer programme (rising from 66.8% to 96.8%) and completion of ≥4 ANC visits (rising from 16.9% to 43.1%). These trends were statistically significant according to both Cuzick’s non-parametric test for trend (Cuzick z=30.02 and 26.94, respectively; both p<0.001) and Kendall’s τ correlation coefficients (Kendall’s τ-b=0.15 and 0.14, respectively; both p<0.001), indicating an improvement in both metrics over time.

### Results: reproductive history-related factors

Women with mildly preterm (34–37 weeks) and moderately preterm (<34 weeks) births were less likely to have attended ≥4 ANC visits compared with term births (adjusted OR=0.67, 95% CI (0.61 to 0.73)) for mildly preterm; and for moderately preterm (adjusted OR=0.41, 95% CI (0.35 to 0.49)). Women with ≥1 parity were less likely to attend ≥4 ANC visits than those with no prior pregnancies (adjusted OR=0.66, 95% CI (0.61 to 0.72)). Similarly, those with a previous pregnancy but no prior LB were more likely to attend ≥4 ANC visits than those with a previous LB (adjusted OR=2.14, 95% CI (1.57 to 2.92)). Women with an IPI of >36 months were more likely to attend ≥4 ANC visits compared with those with an IPI of 18–36 months (OR=1.31, 95% CI (1.19 to 1.45)).

### Results: morbidity during pregnancy

Women who experienced hypertension at least once during their second or third trimesters were less likely to have attended ≥4 ANC visits compared with those who did not experience hypertension (adjusted OR=0.79, 95% CI (0.69 to 0.90)). Pregnancy-related morbidities, including respiratory problems, poor appetite, vaginal bleeding, swelling of hands and face and STIs, were associated with statistically significant increased odds of ≥4 ANC visits and respective ORs are listed in table 2.

## Discussion

We observed multilevel factors associated with the receipt of ≥4 ANC visits. These factors included SES and demographic characteristics (eg, non-farming occupations for women, higher education, wealth quintile, maternal height and age, caste), and women’s awareness of the conditional cash transfer programme. Additionally, pregnancy history and pregnancy-related morbidities such as first-time pregnant women, gestational age at delivery, IPIs exceeding 36 months, prior pregnancy but no LB, and preterm birth were associated with the likelihood of achieving ≥4 ANC visits. Also, women with morbidity symptoms in pregnancy, including respiratory issues, swelling of the hands and face, vaginal bleeding, poor appetite, nausea, vomiting or an STI, were more likely to attend ≥4 ANC visits.

We found that women engaged in business, private or government services had a higher likelihood of receiving ≥4 ANC visits, as did women with husbands in non-agricultural sectors. This suggests that household members’ occupations, indicative of their financial situation, influence women’s access and utilisation of healthcare services during pregnancy in Nepal.

Previous research has shown that higher education of husbands, with their increased involvement during their wives’ pregnancies, could increase the likelihood of women attending ≥4 ANC visits, thereby benefiting maternal health.^14^ Our study found that women whose husbands were employed in non-agricultural sectors were more likely to attend ≥4 ANC visits. This indicates the role husbands play in maternal health and ANC, independent of the wife’s educational background. However, we were not able to examine the role of other family members such as the mother-in-law in influencing ANC visits.

We found a positive dose-response between higher educational levels and the receipt of ≥4 ANC visits. Prior studies using NDHS 2001, 2011 and 2016 data align with our findings, emphasising the role of education in facilitating the use of maternal health services and the positive associations with higher number of ANC visits.^8 13 22^ Higher education enhances women’s understanding of the importance of professional healthcare, broadening their accessibility and utilisation.^14 23^ In Nepal, the adult literacy rate for women is 60%, lower than the 68% recorded for men.^24^ Literacy rates in NOMS were much lower than for Nepal as a whole.^6^ Promoting education, particularly for girls, might increase ANC utilisation. Health education campaigns targeted at illiterate women with financial incentives may be short-term interventions that improve ANC attendances.^22^ Longer-term interventions such as advocating for women’s education and employment may increase ANC attendance and overall maternal and neonatal health in Nepal.^25^ In our study, women from wealthier households were more likely to have attended ≥4 ANC visits, which is consistent with results from previous studies.^14 19 22^ Therefore, there is a need to tailor ANC services to better support women from poor and less educated backgrounds to promote more attendance of ANC care.

Caste was associated with the receipt of ≥4 ANC visits. In Nepal, Brahmin and Chhetri are considered to be of higher caste than Vaishya and Shudra.^26^ Our findings suggest that Brahmin and Chhetri women were more likely to attend ≥4 ANC visits compared with those identified as Vaishya, Shudra or Muslim. Similarly, another study analysed 2014–2019 national survey data and found that women from Dalit, Disadvantaged Janajati and Other Madheshi groups were less likely to complete recommended ANC visits than Brahmin and Chhetri.^19^

Maternal age was associated with 4+ ANC visits. Women under 18 or over 35 years of age were less likely to have ≥4 visits, independent of parity, although younger women were more likely to be parity zero and older women multiparous. Our results suggest that maternal height was independently associated with ANC attendance. Specifically, taller women were more inclined to have had at least four ANC visits, compared with women shorter than 145 cm. Prior studies suggest that shorter women had greater risk of preterm delivery, delivering low birth weight babies and neonatal mortality, which indicated maternal height as a direct determinant of adverse birth outcomes.^5 12 27 28^

Awareness of the government conditional cash transfer programme influenced the likelihood of attending ≥4 ANC visits. The Aama Surakshya Programme (conditional cash transfer programme) was a cash-based incentive programme introduced by the Government of Nepal (GoN) to increase ANC attendance, promote institutional deliveries and reduce maternal morbidity and mortality.^29^ The GoN reported that the Aama programme had a positive impact on institutional deliveries for pregnant women in Nepal in 2020, with a strong relationship between the programme and completing ≥4 ANC visits.^29^ Our results suggested that the odds of ≥4 ANC visits increased from 2010 through 2017, which likely reflects that more women knew about the conditional cash transfer programme and made more use of it over this time period. Therefore, it is important to continue promoting the conditional cash transfer programme through methods such as enhancing government financial support, improving community advocacy and engagement among marginalised women.

Our study found that first-time pregnant women were more likely to have ≥4 ANC visits compared with those with parity ≥1. Our finding is inconsistent with a prior study from Western Nepal, which found that primiparous women had lower probability of receiving the recommended number of ANC visits.^18^ A population-based study using 2011 Nepal DHS data found that the odds of receiving good quality ANC decreased by 21% for every additional pregnancy that resulted in a live or SB.^14^ The number of ANC visits might be associated with the continuity of ANC, which was a component of the quality of ANC. We observed that women with prior SB histories might be more likely to attend ≥4 ANC visits, although not statistically significant, which may be due to the low prevalence of prior SBs (4.3%) in our study. Additionally, the result suggested that women with prior pregnancy but no LB were more likely to receive ≥4 ANC visits relative to women with prior LB pregnancy. It is possible that fear or concern due to past experiences of adverse birth outcomes may increase the receipt of ≥4 ANC visits.

We identified gestational age at delivery as a factor associated with attending ≥4 ANC visits. In Nepal, the suggested schedule for the four ANC visits is distributed across the fourth (12–16 weeks), sixth (20–24 weeks), eighth (28–32 weeks) and ninth (36–40 weeks) months of pregnancy.^21^ Consequently, a pregnancy culminating at 33 weeks or less would reduce the likelihood of attending four or more ANC visits, especially given that the fourth ANC visit is typically scheduled for the ninth month. Prior studies indicate that high-quality ANC is important in mitigating preterm labour.^14^,^16^ Thus, efforts in prompting women to attend more ANC, coupled with enhancements in quality of ANC care, may reduce preterm birth. However, we did not measure the quality of the ANC in this study, only receipt of care.

We also found that women with IPIs exceeding 36 months were more likely to have attended ≥4 ANC visits. One reason may be that women with longer IPIs may perceive their current pregnancy to be higher risk. Alternatively, longer IPIs may be associated with suboptimal health of these women, prompting them to seek increased ANC. A study conducted in Nepal found that both very short IPIs (<18 months) and longer IPIs (beyond 59 months) were associated with higher risks of poor maternal and perinatal outcomes.^25^

In our study, women who had multiple births were more likely to attend ≥4 ANC visits. However, given the few multiple gestational pregnancies in our sample (n=256, representing 0.8%), this association was not statistically significant. One possible explanation is that if women were informed about their multiple gestation, they might be more proactive in seeking ANC. Although rare in this study, ultrasound is becoming more common in this area, and relatively early knowledge of multiple gestation may be increasing.^30^

Our study indicates that during the second or third trimesters, women who reported any of the following symptoms: respiratory issues, swelling of the hands and face, vaginal bleeding, symptoms like poor appetite, nausea, vomiting or an STI, were more likely to attend ≥4 ANC visits. Women with signs and symptoms of morbidity would be more likely to seek care in pregnancy more frequently than those who feel healthy. In addition, the NOMS study protocol directed study workers to refer women reporting certain gestational morbidity symptoms to health facilities, though compliance with this referral was not tracked in our data. Conversely, women who had at least one hypertensive measurement were less likely to attend ≥4 ANC visits. Even though women were told by the study team if they had hypertensive measurements, given that this is often without noticeable symptoms, affected women might not perceive an immediate need to seek further care.

### Strengths and limitations

One of the strengths of our study was the utilisation of a population-based pregnancy trial with a large sample size, enabling a comprehensive examination of potential multilevel risk factors measured prospectively throughout pregnancies. However, due to the limitations with observational studies, establishing a causal relationship between the factors discussed and the attendance of four or more ANC visits remains challenging. In addition, this was a trial in which the entire population of this geographic area was surveyed for pregnancy over the study period. Houses with women of childbearing age were visited every 5 weeks, and any women who missed a period were tested for pregnancy.^6^ With the exception of a very small number of women who refused participation, this was representative of all women who became pregnant during the time period in that geographic area.^6^ Also, this is an area very similar to rural populations in the plains of northern India, southern Nepal, western Bangladesh and southern and central Pakistan.^6^ However, the external generalisability may be limited given that factors contributing to the receipt of ≥4 ANC visits, particularly in urban areas or places where the healthcare system varies, might be different. Also, there may be residual confounding from unmeasured medical conditions during follow-up visits. We also asked the question about the number of ANC visits with the exact wording used in the Nepal DHS. However, another study in this setting found that the validity of maternal recall of the number of ANC visits at 6 months postpartum is poor, although we do not know its validity when being asked at the time of delivery as in our study.^31^ Recall bias is a potential limitation, as certain factors were ascertained through retrospective questioning. Most of our information is based on self-reported answers, introducing the possibility of response biases, including social desirability bias. The number of ANC visits was collected prospectively, but there was no evaluation of ANC quality. The presence of missing data for certain characteristics could introduce bias. Specifically, we did not have much morbidity data during the first trimester because it was difficult to identify and enrol women so early in pregnancy. Hence, first trimester morbidity was excluded from the morbidity analysis.

## Conclusions

In summary, multiple SES factors are associated with a higher probability of the receipt of ≥4 ANC visits in Nepal. Our identification of factors associated with attendance of ≥4 ANC visits in Nepal may help design programmes to increase ANC uptake. The 2022 NDHS reports that 81% women received ≥4 ANC visits and 94% women had at least one ANC from a skilled provider.^4^ Given that only 28% of pregnancies in our study reported ≥4 ANC visits, there is significant potential for improvement in this ANC coverage. Specifically, programmes such as the Aama Surakshya have increased ANC attendance, independent of SES factors. Therefore, it is important to scale up awareness and easy enrolment for the conditional cash transfer programme in Nepal. For example, launching a campaign to increase understanding of the cash incentives programme, communicating the conditions (completing 4+ ANC visits), the amount, and the process for receiving the cash transfer, and simplifying eligibility checks. Also, it is necessary to use diverse channels to promote the programme, including local leaders, mobile phone messages and posters at high-traffic areas. We did not examine the quality of ANC nor whether attending four or more ANC visits led to improved outcomes. However, assuming that more ANC visits are beneficial,^5^ promotion of the conditional cash transfer programme would likely increase ANC attendance and improve maternal and neonatal health in the long term.

Our results suggest that women and their husbands who were unskilled, day or contracted labourers, women from the lowest wealth quintile, less educated, whose height was below 145 cm, who identified as Vaishya, Shudra and Muslim/other, should be prioritised with targeted methods (eg, women’s supporting groups, community leaders), transportation vouchers and fee waivers. Additionally, pregnant women <18 years or >35 years old, mothers of preterm babies and multiparous women should be provided with flexible appointment hours, privacy-sensitive counselling and re-booking options at postnatal discharge to ensure earlier engagement in subsequent pregnancies. Educating pregnant women about morbidity signs and symptoms during pregnancy may increase ANC service utilisation and possibly reduce maternal and neonatal mortality.

## Supplementary material

10.1136/bmjopen-2024-098478 online supplemental file 1

## Data Availability

Data are available upon reasonable request.
