# Supplementary material for: Observational analysis of factors associated with completion of four or more antenatal care visits in Sarlahi district, Nepal
Source: BMJ Open. 2026 Mar 10;16(3):e098478. doi: 10.1136/bmjopen-2024-098478 (PMC12983749; doi:10.1136/bmjopen-2024-098478)
Supplement: online supplemental file 1 [file bmjopen-16-3-s001.docx]

**Supplementary Table 1. Explanations of Covariates**

| **Covariates** | **Explanations** |
| --- | --- |
| ***Women/Husband occupation*** | Occupations of women and their husbands were each categorized into four groups: “Farmer” (reference), “Does not work outside home”, “Unskilled laborer/day labor or contracted laborer", and "Business or private or government service". |
| ***Maternal age (years)*** | Maternal age groups were: 18 to 35 (reference), below 18, and over 35. |
| ***Maternal height (centimeters)*** | Maternal height was categorized into 3 groups: <145 cm (reference), 145-150 cm, and ≥150 cm. |
| ***Caste/Religion*** | Caste/religion was categorized into 4 groups: Brahmin and Chhetri (reference), Vaishya, Shudra, and Muslim and others, with Brahmin and Chhetri being the two highest castes for those of the Hindu religion. |
| ***Mother's education (years)*** | Maternal education was divided into 3 groups: No schooling (reference), 1 to 5 years, and more than 5 years. |
| ***Wealth quintile*** | Wealth was classified into 5 quintiles, ranging from poorest, poorer, middle, richer to richest. The wealth index was constructed from ownership of land and household goods, house construction, latrine type and water source, and whether the home had electricity. |
| ***Gestational age (weeks)*** | Gestational age was categorized into 3 groups: "Moderately preterm: <34 weeks", "Mildly preterm: ≥34-37 weeks" and "Term: ≥37 weeks". |
| ***Multiple birth*** | Multiple birth was a binary variable: singleton (reference), and twin or triplet. |
| ***Parity*** | Parity denoted the number of times that a woman had given birth to a baby of at least 28 weeks of gestational age, regardless of whether it was a live birth or stillbirth. The parity variable “parity including both live birth and stillbirth at enrollment” was categorized into 3 groups: "No Prior Pregnancy (reference)", "Prior pregnancy but no live or stillbirth", and "Parity ≥1". |
| ***Interpregnancy interval (months)*** | Interpregnancy Interval was categorized into 4 groups: 18 to 36 months (reference), less than 18 months, more than 36 months, and no prior pregnancy. |
| ***Any Prior live birth (LB) died*** | Any prior LB died was categorized as 4 groups: “Prior LB but not died” (reference), “Prior LB died”, “Prior Pregnancy but no LB”, and “No prior pregnancy”. |
| ***Any prior pregnancy ended in stillbirth (SB)*** | Any prior pregnancy ended in SB was categorized into 3 groups: “Prior pregnancy but no SB” (reference), “Prior SB”, and “No prior pregnancy”. |
| ***Knowledge of conditional cash transfer program*** | Awareness about the “Conditional cash transfer program” was a binary response, indicating whether the woman was familiar with the government’s conditional cash transfer program that pays women if they have 4 or more ANC visits and an additional payment for delivery at a facility. |
| ***Year of childbirth*** | “Year of birth of the child” indicated the exact year of the birth of the child during each pregnancy, ranging from 2010 to 2017. |
| ***Morbidity indicators*** | |
| ***Fever*** | Fever was defined as a temperature of at least 99 Fahrenheit during the second or third trimesters, recorded during monthly visits. |
| ***Tachycardia*** | Tachycardia referred to a pulse rate of 100 beats per minute or higher measured at monthly visits during these trimesters. |
| ***Hypertension*** | Hypertension was identified by a systolic blood pressure above 140 mmHg or a diastolic pressure above 90 mmHg in the latter two trimesters. |
| ***Poor appetite, nausea & vomiting*** | Upper gastrointestinal (GI) symptoms included poor appetite, vomiting, and nausea, while lower GI symptoms involved watery stools or blood/mucus presence. |
| ***Respiratory problem*** | Respiratory illness included symptoms like coughing, breathlessness, wheezing, or shortness of breath. |
| ***Swelling of hands or face*** | Swelling symptoms referred only to swelling of hands and face to indicate possible pre-eclampsia; leg and foot swelling was omitted due to it being common in pregnancy. |
| ***Vaginal bleeding*** | Vaginal bleeding meant any bleeding during pregnancy. |
| ***Sexually transmitted illness (STI)*** | STI was identified by painful urination or a foul-smelling vaginal discharge. |

Note: All mentioned morbidity indicators were binary and based on maternal recall in the past 30 days, except for blood pressure, pulse and temperature, which were measured at each monthly visit during pregnancy.

**Supplementary Table 2. Longitudinal risk factors for ≥3 ANC visits**

|  | **Crude Odds Ratio** | **95% CI** | **Adjusted Odds Ratio** | **95% CI** |
| --- | --- | --- | --- | --- |
| ***Women occupation*** |  |  |  |  |
| Farmer | Reference | Reference | Reference | Reference |
| Does not work | **1.33**** | **1.20, 1.47** | 0.94 | 0.84, 1.05 |
| Unskilled/day labor/contracted laborer | **0.80*** | **0.67, 0.95** | 0.95 | 0.78, 1.16 |
| Business/private/government service | **3.10**** | **2.46, 3.91** | **1.48**** | **1.16, 1.89** |
| ***Husband occupation*** |  |  |  |  |
| Farmer | Reference | Reference | Reference | Reference |
| Does not work | **1.46**** | **1.26, 1.70** | 1.18 | 1.00, 1.39 |
| Unskilled laborer/day labor or contracted laborer | **0.94*** | **0.88, 0.99** | **1.07*** | **1.00, 1.15** |
| Business/private service/government  service | **1.49**** | **1.40, 1.60** | **1.24**** | **1.15, 1.33** |
| ***Maternal age (years)*** |  |  |  |  |
| 18-35 | Reference | Reference | Reference | Reference |
| <18 | 0.98 | 0.92, 1.04 | **0.79**** | **0.73, 0.86** |
| >35 | **0.63**** | **0.54, 0.74** | **0.80*** | **0.66, 0.97** |
| ***Maternal height (cm)*** |  |  |  |  |
| <145 | Reference | Reference | Reference | Reference |
| 145-150 | **1.23**** | **1.14, 1.33** | **1.13**** | **1.04, 1.23** |
| ≥150 | **1.38**** | **1.29, 1.49** | **1.15**** | **1.06, 1.24** |
| ***Caste/ethnicity*** |  |  |  |  |
| Brahmin and Chhetri | Reference | Reference | Reference | Reference |
| Vaishya | **0.59**** | **0.51, 0.68** | **0.75**** | **0.64, 0.88** |
| Shudra | **0.41**** | **0.35, 0.47** | **0.66**** | **0.55, 0.78** |
| Muslim and others | **0.55**** | **0.47, 0.65** | **0.76**** | **0.64, 0.91** |
| ***Women education (years)*** |  |  |  |  |
| No schooling | Reference | Reference | Reference | Reference |
| 1-5 | **1.64**** | **1.51, 1.79** | **1.40**** | **1.28, 1.54** |
| >5 | **2.76**** | **2.59, 2.93** | **1.93**** | **1.80, 2.08** |
| ***Wealth quintile*** |  |  |  |  |
| Poorest | Reference | Reference | Reference | Reference |
| Poorer | **1.40**** | **1.31, 1.51** | **1.20**** | **1.11, 1.30** |
| Middle | **1.67**** | **1.55, 1.80** | **1.32**** | **1.21, 1.43** |
| Richer | **1.96**** | **1.82, 2.11** | **1.33**** | **1.22, 1.46** |
| Richest | **2.49**** | **2.30, 2.69** | **1.39**** | **1.26, 1.53** |
| ***Gestational age (weeks)*** |  |  |  |  |
| Term (≥37) | Reference | Reference | Reference | Reference |
| Mildly preterm (34-37) | **0.74**** | **0.69, 0.80** | **0.79**** | **0.73, 0.85** |
| Moderately preterm (<34) | **0.47**** | **0.41, 0.52** | **0.51**** | **0.44, 0.58** |
| ***Multiple birth*** |  |  |  |  |
| Singleton | Reference | Reference | Reference | Reference |
| Twin/Triplet | 0.86 | 0.66, 1.11 | 1.25 | 0.94, 1.66 |
| ***Parity*** |  |  |  |  |
| No Prior Pregnancy | Reference | Reference | Reference | Reference |
| Prior pregnancy but no live or stillbirth | **1.30**** | **1.11, 1.53** | **0.52**** | **0.37, 0.74** |
| Parity ≥1 | **0.67**** | **0.64, 0.71** | **0.63**** | **0.59, 0.68** |
| ***Interpregnancy Interval (months)*** |  |  |  |  |
| 18-36 | Reference | Reference | Reference | Reference |
| <18 | 1.00 | 0.95, 1.06 | 0.97 | 0.91, 1.04 |
| >36 | **1.27**** | **1.17, 1.38** | **1.22**** | **1.12, 1.34** |
| No prior pregnancy | **1.51**** | **1.42, 1.61** | - | - |
| ***Prior live birth (LB) died*** |  |  |  |  |
| Prior LB but not died | Reference | Reference | Reference | Reference |
| Prior LB died | **0.89**** | **0.83, 0.97** | 1.02 | 0.94, 1.11 |
| Prior pregnancy but no LB | **2.06**** | **1.80, 2.36** | **2.61**** | **1.92, 3.55** |
| No prior pregnancy | **1.48**** | **1.41, 1.56** | - | - |
| ***Prior pregnancy ended in stillbirth (SB)*** |  |  |  |  |
| Prior pregnancy but no SB | Reference | Reference | Reference | Reference |
| Prior SB | **1.17**** | **1.04, 1.31** | 1.11 | 0.96, 1.29 |
| No prior pregnancy | **1.47**** | **1.40, 1.55** | - | - |
| ***Conditional cash transfer program knowledge*** | **2.57**** | **2.36, 2.80** | **2.01**** | **1.83, 2.21** |
| ***Year of childbirth*** | **1.20**** | **1.18, 1.21** | **1.18**** | **1.16, 1.20** |
| ***Fever*** |  |  |  |  |
| No | Reference | Reference | Reference | Reference |
| Yes | 0.98 | 0.91, 1.06 | 1.04 | 0.96, 1.13 |
| ***Tachycardia*** |  |  |  |  |
| No | Reference | Reference | Reference | Reference |
| Yes | 1.01 | 0.97, 1.06 | 0.98 | 0.93, 1.03 |
| ***Hypertension*** |  |  |  |  |
| No | Reference | Reference | Reference | Reference |
| Yes | 0.90 | 0.81, 1.00 | 0.92 | 0.82, 1.04 |
| ***Respiratory problem*** |  |  |  |  |
| No | Reference | Reference | Reference | Reference |
| Yes | **1.19**** | **1.14, 1.25** | **1.14**** | **1.08, 1.21** |
| ***Poor appetite, nausea & vomiting*** |  |  |  |  |
| No | Reference | Reference | Reference | Reference |
| Yes | **1.29**** | **1.23, 1.35** | **1.17**** | **1.11, 1.23** |
| ***Vaginal bleeding*** |  |  |  |  |
| No | Reference | Reference | Reference | Reference |
| Yes | **1.34**** | **1.12, 1.60** | **1.31**** | **1.07, 1.59** |
| ***Swelling of hands and face*** |  |  |  |  |
| No | Reference | Reference | Reference | Reference |
| Yes | **1.30**** | **1.17, 1.44** | **1.30**** | **1.16, 1.45** |
| ***Sexually transmitted illness*** |  |  |  |  |
| No | Reference | Reference | Reference | Reference |
| Yes | **1.22**** | **1.15, 1.29** | **1.26**** | **1.19, 1.34** |

Note: ** p<0.01, * p<0.05

*Morbidity symptoms in pregnancy are for presence at any time in either the 2nd and/or 3rd trimesters

**Supplementary Table 3. Longitudinal risk factors for ≥5 ANC visits**

|  | **Crude Odds Ratio** | **95% CI** | **Adjusted Odds Ratio** | **95% CI** |
| --- | --- | --- | --- | --- |
| ***Women occupation*** |  |  |  |  |
| Farmer | Reference | Reference | Reference | Reference |
| Does not work | **1.26*** | **1.03, 1.54** | 0.84 | 0.68, 1.05 |
| Unskilled/day labor/contracted laborer | **0.48**** | **0.31, 0.75** | 0.73 | 0.45, 1.18 |
| Business/private/government service | **3.72**** | **2.71, 5.10** | **1.48*** | **1.05, 2.08** |
| ***Husband occupation*** |  |  |  |  |
| Farmer | Reference | Reference | Reference | Reference |
| Does not work | **1.49**** | **1.15, 1.93** | 1.05 | 0.80, 1.38 |
| Unskilled laborer/day labor or contracted laborer | **0.88*** | **0.78, 0.98** | 1.10 | 0.97, 1.25 |
| Business/private service/government  service | **1.89**** | **1.68, 2.12** | **1.51**** | **1.33, 1.70** |
| ***Maternal age (years)*** |  |  |  |  |
| 18-35 | Reference | Reference | Reference | Reference |
| <18 | 1.00 | 0.88, 1.12 | **0.81**** | **0.71, 0.94** |
| >35 | **0.61**** | **0.43, 0.87** | 0.95 | 0.66, 1.37 |
| ***Maternal height (cm)*** |  |  |  |  |
| <145 | Reference | Reference | Reference | Reference |
| 145-150 | **1.32**** | **1.13, 1.55** | 1.17 | 0.99, 1.38 |
| ≥150 | **1.67**** | **1.44, 1.93** | **1.27**** | **1.09, 1.49** |
| ***Caste/ethnicity*** |  |  |  |  |
| Brahmin and Chhetri | Reference | Reference | Reference | Reference |
| Vaishya | **0.41**** | **0.34, 0.49** | **0.59**** | **0.48, 0.71** |
| Shudra | **0.24**** | **0.19, 0.29** | **0.48**** | **0.37, 0.62** |
| Muslim and others | **0.38**** | **0.30, 0.47** | **0.63**** | **0.49, 0.81** |
| ***Women education (years)*** |  |  |  |  |
| No schooling | Reference | Reference | Reference | Reference |
| 1-5 | **1.79**** | **1.54, 2.09** | **1.44**** | **1.22, 1.70** |
| >5 | **3.22**** | **2.93, 3.53** | **1.92**** | **1.71, 2.16** |
| ***Wealth quintile*** |  |  |  |  |
| Poorest | Reference | Reference | Reference | Reference |
| Poorer | **1.40**** | **1.18, 1.65** | 1.13 | 0.95, 1.35 |
| Middle | **1.60**** | **1.36, 1.88** | 1.13 | 0.94, 1.35 |
| Richer | **2.35**** | **2.02, 2.74** | **1.39**** | **1.16, 1.66** |
| Richest | **3.28**** | **2.83, 3.80** | **1.49**** | **1.24, 1.79** |
| ***Gestational age (weeks)*** |  |  |  |  |
| Term (≥37) | Reference | Reference | Reference | Reference |
| Mildly preterm (34-37) | **0.73**** | **0.62, 0.84** | **0.79**** | **0.67, 0.93** |
| Moderately preterm (<34) | **0.42**** | **0.31, 0.56** | **0.49**** | **0.35, 0.67** |
| ***Multiple birth*** |  |  |  |  |
| Singleton | Reference | Reference | Reference | Reference |
| Twin/Triplet | 1.36 | 0.90, 2.07 | **1.94**** | **1.21, 3.09** |
| ***Parity*** |  |  |  |  |
| No Prior Pregnancy | Reference | Reference | Reference | Reference |
| Prior pregnancy but no live or stillbirth | **1.81**** | **1.47, 2.23** | **0.58*** | **0.35, 0.98** |
| Parity ≥1 | **0.58**** | **0.53, 0.64** | **0.60**** | **0.52, 0.68** |
| ***Interpregnancy Interval (months)*** |  |  |  |  |
| 18-36 | Reference | Reference | Reference | Reference |
| <18 | 1.05 | 0.93, 1.18 | 0.93 | 0.82, 1.06 |
| >36 | **1.49**** | **1.28, 1.75** | **1.37**** | **1.16, 1.61** |
| No prior pregnancy | **1.77**** | **1.57, 1.99** | - | - |
| ***Prior live birth (LB) died*** |  |  |  |  |
| Prior LB but not died | Reference | Reference | Reference | Reference |
| Prior LB died | 0.99 | 0.84, 1.15 | **1.19*** | **1.01, 1.40** |
| Prior pregnancy but no LB | **3.24**** | **2.71, 3.87** | **3.44**** | **2.17, 5.45** |
| No prior pregnancy | **1.76**** | **1.60, 1.93** | - | - |
| ***Prior pregnancy ended in stillbirth (SB)*** |  |  |  |  |
| Prior pregnancy but no SB | Reference | Reference | Reference | Reference |
| Prior SB | **1.25*** | **1.01, 1.54** | 1.06 | 0.78, 1.46 |
| No prior pregnancy | **1.64**** | **1.50, 1.79** | - | - |
| ***Conditional cash transfer program knowledge*** | **2.34**** | **1.91, 2.88** | **1.60**** | **1.29, 2.00** |
| ***Year of childbirth*** | **1.24**** | **1.21, 1.28** | **1.21**** | **1.18, 1.25** |
| ***Fever*** |  |  |  |  |
| No | Reference | Reference | Reference | Reference |
| Yes | 0.88 | 0.76, 1.01 | 0.96 | 0.83, 1.12 |
| ***Tachycardia*** |  |  |  |  |
| No | Reference | Reference | Reference | Reference |
| Yes | 0.98 | 0.90, 1.07 | 0.97 | 0.89, 1.07 |
| ***Hypertension*** |  |  |  |  |
| No | Reference | Reference | Reference | Reference |
| Yes | 0.84 | 0.67, 1.04 | 0.83 | 0.66, 1.06 |
| ***Respiratory problem*** |  |  |  |  |
| No | Reference | Reference | Reference | Reference |
| Yes | **1.16**** | **1.06, 1.27** | 1.10 | 1.00, 1.21 |
| ***Poor appetite, nausea & vomiting*** |  |  |  |  |
| No | Reference | Reference | Reference | Reference |
| Yes | **1.26**** | **1.16, 1.38** | **1.14**** | **1.04, 1.26** |
| ***Vaginal bleeding*** |  |  |  |  |
| No | Reference | Reference | Reference | Reference |
| Yes | **1.76**** | **1.35, 2.29** | **1.80**** | **1.36, 2.38** |
| ***Swelling of hands and face*** |  |  |  |  |
| No | Reference | Reference | Reference | Reference |
| Yes | **1.44**** | **1.21, 1.70** | **1.45**** | **1.21, 1.74** |
| ***Sexually transmitted illness*** |  |  |  |  |
| No | Reference | Reference | Reference | Reference |
| Yes | **1.25**** | **1.13, 1.38** | **1.27**** | **1.14, 1.41** |

Note: ** p<0.01, * p<0.05

*Morbidity symptoms in pregnancy are for presence at any time in either the 2nd and/or 3rd trimesters

**Supplementary Table 4. Longitudinal risk factors for ≥8 ANC visits**

|  | **Crude Odds Ratio** | **95% CI** | **Adjusted Odds Ratio** | **95% CI** |
| --- | --- | --- | --- | --- |
| ***Women occupation*** |  |  |  |  |
| Farmer | Reference | Reference | Reference | Reference |
| Does not work | 1.67 | 0.92, 3.05 | 1.44 | 0.73, 2.86 |
| Unskilled/day labor/contracted laborer | 0.39 | 0.09, 1.73 | 0.85 | 0.18, 4.03 |
| Business/private/government service | **5.11**** | **2.31, 11.32** | 2.26 | 0.96, 5.36 |
| ***Husband occupation*** |  |  |  |  |
| Farmer | Reference | Reference | Reference | Reference |
| Does not work | 1.66 | 0.90, 3.07 | 0.97 | 0.51, 1.85 |
| Unskilled laborer/day labor or contracted laborer | 0.87 | 0.65, 1.16 | 1.21 | 0.89, 1.64 |
| Business/private service/government  service | **1.94**** | **1.46, 2.58** | **1.35*** | **1.01, 1.80** |
| ***Maternal age (years)*** |  |  |  |  |
| 18-35 | Reference | Reference | Reference | Reference |
| <18 | 0.77 | 0.55, 1.07 | **0.62**** | **0.43, 0.88** |
| >35 | 1.12 | 0.57, 2.18 | 1.95 | 0.92, 4.12 |
| ***Maternal height (cm)*** |  |  |  |  |
| <145 | Reference | Reference | Reference | Reference |
| 145-150 | **1.69*** | **1.07, 2.66** | 1.50 | 0.93, 2.42 |
| ≥150 | **2.52**** | **1.65, 3.84** | **1.89**** | **1.20, 2.96** |
| ***Caste/ethnicity*** |  |  |  |  |
| Brahmin and Chhetri | Reference | Reference | Reference | Reference |
| Vaishya | **0.29**** | **0.20, 0.42** | **0.42**** | **0.28, 0.61** |
| Shudra | **0.14**** | **0.09, 0.21** | **0.35**** | **0.20, 0.61** |
| Muslim and others | **0.27**** | **0.17, 0.45** | **0.47**** | **0.27, 0.82** |
| ***Women education (years)*** |  |  |  |  |
| No schooling | Reference | Reference | Reference | Reference |
| 1-5 | **1.96**** | **1.31, 2.92** | **1.59*** | **1.05, 2.42** |
| >5 | **4.21**** | **3.34, 5.32** | **2.35**** | **1.74, 3.18** |
| ***Wealth quintile*** |  |  |  |  |
| Poorest | Reference | Reference | Reference | Reference |
| Poorer | 1.55 | 0.97, 2.48 | 1.22 | 0.74, 2.01 |
| Middle | **1.73*** | **1.09, 2.74** | 1.17 | 0.71, 1.92 |
| Richer | **3.06**** | **2.00, 4.67** | **1.66*** | **1.03, 2.67** |
| Richest | **4.70**** | **3.14, 7.05** | **1.87*** | **1.16, 3.03** |
| ***Gestational age (weeks)*** |  |  |  |  |
| Term (≥37) | Reference | Reference | Reference | Reference |
| Mildly preterm (34-37) | 0.67 | 0.45, 1.01 | 0.75 | 0.49, 1.15 |
| Moderately preterm (<34) | 0.77 | 0.42, 1.40 | 0.87 | 0.45, 1.71 |
| ***Multiple birth*** |  |  |  |  |
| Singleton | Reference | Reference | Reference | Reference |
| Twin/Triplet | 1.91 | 0.79, 4.62 | 2.17 | 0.77, 6.10 |
| ***Parity*** |  |  |  |  |
| No Prior Pregnancy | Reference | Reference | Reference | Reference |
| Prior pregnancy but no live or stillbirth | **3.00**** | **2.01, 4.47** | 0.65 | 0.19, 2.28 |
| Parity ≥1 | **0.57**** | **0.45, 0.72** | **0.55**** | **0.39, 0.77** |
| ***Interpregnancy Interval (months)*** |  |  |  |  |
| 18-36 | Reference | Reference | Reference | Reference |
| <18 | 1.21 | 0.89, 1.65 | 0.93 | 0.66, 1.30 |
| >36 | 1.50 | 0.99, 2.25 | 1.22 | 0.79, 1.89 |
| No prior pregnancy | **1.81**** | **1.33, 2.45** | - | - |
| ***Prior live birth (LB) died*** |  |  |  |  |
| Prior LB but not died | Reference | Reference | Reference | Reference |
| Prior LB died | 1.36 | 0.93, 1.98 | **1.78**** | **1.20, 2.65** |
| Prior pregnancy but no LB | **5.81**** | **4.06, 8.31** | **5.15**** | **1.64, 16.22** |
| No prior pregnancy | **1.98**** | **1.54, 2.55** | - | - |
| ***Prior pregnancy ended in stillbirth (SB)*** |  |  |  |  |
| Prior pregnancy but no SB | Reference | Reference | Reference | Reference |
| Prior SB | 1.39 | 0.83, 2.35 | 1.11 | 0.43, 2.87 |
| No prior pregnancy | **1.58**** | **1.26, 1.99** | - | - |
| ***Conditional cash transfer program knowledge*** | **2.54**** | **1.44, 4.51** | 1.78 | 0.94, 3.37 |
| ***Year of childbirth*** | **1.29**** | **1.20, 1.38** | **1.23**** | **1.14, 1.33** |
| ***Fever*** |  |  |  |  |
| No | Reference | Reference | Reference | Reference |
| Yes | 0.67 | 0.44, 1.01 | 0.80 | 0.52, 1.23 |
| ***Tachycardia*** |  |  |  |  |
| No | Reference | Reference | Reference | Reference |
| Yes | 0.90 | 0.72, 1.12 | 0.91 | 0.73, 1.15 |
| ***Hypertension*** |  |  |  |  |
| No | Reference | Reference | Reference | Reference |
| Yes | 0.66 | 0.35, 1.24 | 0.61 | 0.31, 1.18 |
| ***Respiratory problem*** |  |  |  |  |
| No | Reference | Reference | Reference | Reference |
| Yes | 1.17 | 0.94, 1.46 | 1.08 | 0.85, 1.37 |
| ***Poor appetite, nausea & vomiting*** |  |  |  |  |
| No | Reference | Reference | Reference | Reference |
| Yes | 1.22 | 0.98, 1.51 | 1.09 | 0.86, 1.38 |
| ***Vaginal bleeding*** |  |  |  |  |
| No | Reference | Reference | Reference | Reference |
| Yes | **2.35**** | **1.34, 4.11** | **2.17*** | **1.19, 3.97** |
| ***Swelling of hands and face*** |  |  |  |  |
| No | Reference | Reference | Reference | Reference |
| Yes | **1.78**** | **1.21, 2.60** | **1.56*** | **1.04, 2.34** |
| ***Sexually transmitted illness*** |  |  |  |  |
| No | Reference | Reference | Reference | Reference |
| Yes | **1.29*** | **1.01, 1.65** | 1.28 | 0.98, 1.67 |

Note: ** p<0.01, * p<0.05

*Morbidity symptoms in pregnancy are for presence at any time in either the 2nd and/or 3rd trimesters
