# Supplementary material for: Observational analysis of factors associated with completion of four or more antenatal care visits in Sarlahi district, Nepal
Source: BMJ Open. 2026 Mar 10;16(3):e098478. doi: 10.1136/bmjopen-2024-098478 (PMC12983749; doi:10.1136/bmjopen-2024-098478)
Supplement: Supplementary file 2 [file bmjopen-2024-098478.reviewer_comments.pdf]

PEER REVIEW HISTORY

BMJ Open publishes all reviews undertaken for accepted manuscripts. Reviewers are asked to complete a checklist review form and are provided with free text boxes to elaborate on their assessment. These free text comments are reproduced below.

ARTICLE DETAILS

Title (Provisional)

Observational Analysis of Factors Associated with Completion of Four or More Antenatal Care Visits in Sarlahi District, Nepal.

Authors

Yue, Yiwei; Hazel , Elizabeth A; Subedi, Seema; Zeger, Scott; Mohan, Diwakar; Mullany, Luke C.; Tielsch, James M; Khatry, Subarna; LeClerq, Steven C.; Katz, Joanne

---

VERSION 1 - REVIEW

**Reviewer**	**1**
**Name**	**Geta, Temesgen**
**Affiliation**	**College of Health Science and Medicine Wolaita Sodo University**
**Date**	**12-Feb-2025**
**COI**	**None**

The authors need to address the following comments.

1. Why the authors prepare old model over new WHO 2016 8 contacts.

2. The authors need to revise title of the study including prevalence of ANC or they have to remove prevalence from the whole document.

3. introduction part needs an extensive revision such as Description about ANC 1-4,factors tha affect anc

4. Manuscript has poos conclusion with no recommendations

5.methods needs clear description about study participans like source population , sample size, tools, validity, reliability, eligibility criteria.

6.discussion missed comparison and implications of the findings

7. Authors need to state the gap of the study under abstract and last paragraph of introduction.

8. Keywords are too much. Try to write critical word only

**Reviewer**	**2**
**Name**	**Hoxha, Ilir**
**Affiliation**	**Kolegjin Heimerer**
**Date**	**22-Sep-2025**
**COI**	**None**

Overall…

This is a very well executed and presented study on predictors of ANC visits in Nepal. I have only a few minor comments which you will find below.

Specific comments

Abstract

Looks good intro, methods, outcome

The sentence "This study investigates factors associated with attending ≥ 4 ANC visits in Sarlahi district of southern Nepal" does not sound complete… factors predicting ANC visits?

I don't see how the conclusion part of claim is supported by results of the study… seem to broad… and not sure I I would agree with that linkage… but maybe authors can justify…

"These findings underscore the importance of continuing and promoting the government's program and increasing awareness among women." The other one is good "Moreover, understanding these factors can guide interventions aimed at encouraging ANC uptake in the most vulnerable groups, subsequently reducing maternal-related adverse outcomes in LMICs.

Article strengths

Very well presented… and honest… overview of strengths and limitations…

One of bullets "Utilized a population-based pregnancy cohort dataset," seems confusing. How come? Didn't you have a secondary analysis of a cluster RCT? That's not a cohort design to me!

Background

Seems ok to me

Methods

Particiants' paragraph seems thin… you may also refer to other publications if the participants have been described in previous publications…

Measures and predictors well described… really well done… I miss some rational why they were included…

Stat analysis seem to account for within person correlation. What about within-cluster correlation? Since this is a cluster RCT, this should be taken into account somehow… you need that typical cluster effect estimation and integration in the analysis…

Results

Seem well organized and presented…

Discussion

Seems a bit confusing… would suggest beter organization… maybe start with summary of result… them strenths and limitations… they try to explain findings in context of other findings… then extend to implications and then conclusion… right now it's a bit messy…

**Reviewer**	**3**
**Name**	**Kelson, Zoe**
**Affiliation**	**University of Exeter, Mathematics**
**Date**	**21-Oct-2025**
**COI**	**None**

This study investigates factors associated with attending ≥ 4 ANC visits in Sarlahi district of southern Nepal.

Reviewer comments:

"A secondary analysis was conducted on data from the Nepal Oil Massage Study (NOMS), a cluster-randomized, community-based longitudinal pregnancy cohort study encompassing 34 Village Development Committees"

Can the authors please further comment on whether the analysed cohort in this study can be considered to be representative?

"All pregnancies resulting in a live birth (n=31,867) were included in the model." [Abstract]

and

"The Nepal Oil Massage Study (NOMS), was a cluster-randomized community-based controlled trial that included mothers and their live and stillborn infants in 34 Village Development Committees (VDCs) in Sarlahi District, rural southern Nepal, from 2010 to 2017 (14)."

and

"We excluded pregnancies resulting in fetal loss or records with missing values or stillbirths for pregnancy outcomes, only considering live births for analysis. All pregnancies that resulted in at least one live birth were included, regardless of their intervention assignment."

Can the authors please clarify why only live births were considered in the study?

"A total of 340 geographic clusters were randomized to receive either sunflower seed oil to massage for newborn babies or the standard mustard seed oil (14). Married women of childbearing age were visited for pregnancy surveillance visits every 5 weeks and enrolled – with consent – when pregnant. "

Can the authors please adjust for treatment arm in the analysis?

Furthermore, can original recruitment design, clustering, and stratification variables please be adjusted for in the analyses?

"The number of ANC visits women attended during pregnancy was obtained from maternal recall by study staff during home visits as soon as possible after delivery. Our primary ANC visit outcome was binarized as four or more ANC visits, and less than 4 ANC visits (including no visits). "

The potential for recall bias has been acknowledged by the authors in the study limitations.

Can a sensitivity analysis please be conducted to explore if the same factors are associated with 3 and 5 ANC visits?

"Predictors including demographic, SES, and obstetric and medical history collected at enrollment. Pregnancy information such as morbidity symptoms were collected monthly throughout pregnancy, including morbidity recall and measurement of pulse, temperature and blood pressure. As soon as possible after birth, study staff interviewed the mother about labor and delivery and recorded the pregnancy outcome (e.g., live births, stillbirths, multiple births). Awareness of a government cash transfer program that incentivized women to receive ANC and deliver at a facility were noted through maternal recall. "

Can the authors please confirm if there was any missing covariate data?

"We quantified the association between receipt/attendance of ≥ 4 ANC visits and socioeconomic, demographic, morbidity, and pregnancy history factors using logistic regression; Generalized Estimating Equations were used to account for multiple pregnancies per woman. " [Abstract]

and

"To examine factors associated with the completion of ≥ 4 ANC visits, we fit Generalized Estimating Equation (GEE) models with a logit link and an exchangeable working correlation structure, to account for the within-person correlation arising from including more than one pregnancy for many women in the study. We presented both the crude and adjusted odds ratio (OR) of ≥ 4 ANC visits with the corresponding 95% confidence intervals (CIs)."

Appropriate modelling methods have been applied.

Can the covariates included in the adjusted models please be specified in the Statistical analysis section too?

"A tenth (11.0%, n = 3,517) of pregnancies were missing the reported number of ANC visits. In our study population, accounting for pregnancies with missing number of ANC visits, there were 28.0% with ≥ 4 ANC visits and 61.0% pregnancies with < 4 ANC visits, with the average number of ANC visits in the whole analyses sample being 2.4 ± 1.8."

Can the authors please clarify how this missing outcome data (i.e. missing number of ANC visits) has been accounted for in this assessment of frequencies?

"Among 28,350 pregnancies with non-missing numbers of ANC visits"

Can the authors please confirm whether models have been developed using complete outcome data (i.e. 28,350 pregnancies, with reported ANC numbers)?

Furthermore, can the authors please comment on whether missing number of reported ANCs be considered to be missing at random?

"From 2010 to 2017, women had an increased probability of attending ≥ 4 ANC visits per year (adjusted OR = 1.19, 95% CI [1.17, 1.22]). The prevalence of ≥ 4 ANC visits by child's birth year increased across time as follows: 64 (16.9%) in 2010, 510 (18.1%) in 2011, 714 (23.7%) in 2012, 576 (19.9%) in 2013, 1,836 (31.6%) in 2014, 2,328 (40.8%) in 2015, 2,689 (37.0%) in 2016, and 190 (43.1%) in 2017, not including missing values in the denominator"

and

"The prevalence of awareness of the government's conditional cash transfer program promoting ≥ 4 ANC visits increased over time: 254 (66.8%) in 2010, 2,286 (82.1%) in 2011, 2,472 (84.3%) in 2012, 2,470 (88.1%) in 2013, 5,408 (93.7%) in 2014, 5,400 (94.7%) in 2015, 6,891 (95.1%) in 2016, and 428 (96.8%) in 2017, not including missing values in the denominator. "

Can the authors please consider exploring changes over time statistically, in a formal trend analysis?

"Table 1. Characteristics of the whole analyses sample and stratified by ≥ 4 ANC visits or not "

and

"Table 2. Longitudinal risk factors for ≥ 4 ANC visits"

Can data on lifestyle factors, such as smoking or alcohol, please be considered for inclusion in the study?

"Challenges in establishing causal relationships due to the observational study design and potential confounding factors. • Maternal recall of ANC visits may introduce recall bias, with

validity potentially differing. • Generalizability can be limited, particularly in urban settings where factors influencing receipt of ≥4 ANC visits may differ. • Self-reported data introduces response biases, and the absence of first-trimester morbidity data and ANC quality evaluation may impact findings." [Strengths and Limitations of the Study]

and

"However, due to the limitations with observational studies, establishing a causal relationship between the factors discussed and the attendance of four or more ANC visits remains challenging. Also, we may have confounding such as unmeasured medical conditions during follow-up visits. We also asked the question about number of ANC visits with the exact wording used in the Nepal DHS. However, another study in this setting found that the validity of maternal recall of the number of ANC visits at six months postpartum is poor, although we do not know its validity when being asked at the time of delivery as in our study (24). In addition, the external generalizability may be low for this study given that factors contributing to the receipt of ≥ 4 ANC visits, particularly in urban areas, might be different. Recall bias is a potential limitation, as certain factors were ascertained through retrospective questioning. Most of our information is based on self-reported answers, introducing the possibility of response biases, including social desirability bias. Only the number of ANC visits was collected prospectively, and there was no evaluation of ANC quality. The presence of missing data for certain characteristics could introduce bias. Specifically, we did not have much morbidity data during the first trimester because it was difficult to identify and enroll women so early in pregnancy. Hence first trimester morbidity was excluded from the morbidity analysis."

A discussion on the study limitations is provided by the authors.

Thanks for providing a copy of the STROBE checklist.

VERSION 1 - AUTHOR RESPONSE

Reviewer: 1
Mr. Temesgen Geta, College of Health Science and Medicine Wolaita Sodo University
Comments to the Author:
The authors need to address the following comments.
1. Why the authors prepare old model over new WHO 2016 8 contacts.

Thank you for the question. Our Nepal Oil Massage Study was conducted from 2010 to 2017, as mentioned in the methods section (page 5). During this period, Nepal’s national guideline promoted at least four antenatal care (ANC) visits (e.g., Nepal Demographic and Health Survey 2016), so we defined our outcome as completion of ≥4 ANC visits.

However, we agree that it is important to investigate factors associated with completion of ≥8 ANC visits in light of the new WHO recommendation. We mentioned this in the manuscript (page 8):

"At the time of this study, Nepal's policy was to promote 4 ANC visits in pregnancy, but WHO is now recommending 8 or more contacts during pregnancy (7,21). With changing recommendations, it would be important to examine factors that are associated with ANC attendance at the new recommendation of 8 visits, as well as other cutoffs of ANC visits. Therefore, we also conducted sensitivity analysis to test the associations of factors with the completion of ⩾3, ⩾5, and ⩾8 ANC visits. All analyses were conducted using Stata software version SP 17.0 (StataCorp. 2021)."

The summary GEE regression result table for longitudinal risk factors for ≥8 ANC visits was on Supplementary Table 4 (pages 34-36).

2. The authors need to revise title of the study including prevalence of ANC or they have to remove prevalence from the whole document.

Thank you for this comment. Our primary ANC visit outcome was binarized as four or more ANC visits, and less than 4 ANC visits, rather than prevalence of ANC visits (page 6). The prevalence we used referred to the prevalence of 4 or more ANC visits. However, in order to be clearer, we have revised the title to "Observational Analysis of Factors Associated with Completion of Four or More Antenatal Care Visits in Sarlahi District, Nepal." To reflect study design (page 1).

3. introduction part needs an extensive revision such as Description about ANC 1-4, factors that affect anc

Thank you for this feedback. We have revised our background by adding descriptions on ANC visits and factors linked to ANC visits (pages 4-5):

"Receiving an adequate number of ANC visits is associated with improved maternal and newborn health (5). Specifically, a large community-based cluster-randomized trial, the Nepal Oil Massage Study (NOMS), examined the impact of switching from mustard to sunflower seed oil for newborn massage on all-cause mortality, mortality among preterm babies, and signs of infection in Nepal from 2010 to 2017 (6). Additionally, the NOMS study found that there was an estimated 33% lower neonatal mortality among babies born to women attending at least four ANC sessions (5). During most of the NOMS study period, the Nepal government and the World Health Organization (WHO) recommended at least four ANC visits during pregnancy since 2002 until 2016, and then WHO updated the recommendation to eight ANC visits in 2016 (7–9), but this was not policy in Nepal during the time period of the study."

"Multiple factors have been linked to ANC utilization in Nepal, such as socio-economic and demographic factors (e.g. education, wealth, caste, occupation, ethnicity, age, cash incentives to encourage the completion of ⩾4 visits (18,19). Although there have been national reports on antenatal care coverage and its components (2,4), few studies have examined the prospective associations between multi-level factors and the completion of ⩾4 ANC visits among a large population in Nepal. Furthermore, ANC coverage remains uneven across Nepal and Sarlahi district is considered a poor region in Nepal (2,4,6). Therefore, it is important to identify district-specific factors associated with completing ⩾4 ANC visits, particularly for marginalized populations such as pregnant women in rural Sarlahi

District, Nepal. Additionally, less is known about the associations between pregnancy history and prior pregnancy outcomes with the attendance of ANC visits in Nepal. Therefore, we aimed to assess the association between demographic factors, socioeconomic status (SES), pregnancy history, morbidity symptoms in pregnancy, and the frequency of ANC visits by women in Sarlahi district of Nepal using NOMS data. These findings will inform interventions to increase ANC visits and improve maternal and neonatal health in Nepal and possibly similar LMICs."

4. Manuscript has poos conclusion with no recommendations

Thank you for this suggestion. We have revised our conclusions by adding specific recommendations. Please see the revised sections below (page 15)

"In summary, multiple SES factors are associated with a higher probability of the receipt of ⩾4 ANC visits in Nepal. Our identification of factors associated with attendance of ⩾4 ANC visits in Nepal may help design programs to increase ANC uptake. The 2022 NDHS reports that 81% women received ⩾4 ANC visits and 94% women had at least one ANC from a skilled provider (4). Given that only 28% of pregnancies in our study reported ⩾ 4 ANC visits, there is significant potential for improvement in this ANC coverage. Specifically, programs such as the Aama Surakshya have increased ANC attendance, independent of SES factors. Therefore, it is important to scale up awareness and easy enrollment for the conditional cash transfer program in Nepal. For example, launching a campaign to increase understanding of the cash incentives program, communicating the conditions (completing 4+ ANC visits), the amount, and the process for receiving the cash transfer, and simplifying eligibility checks. Also, it is necessary to utilize diverse channels to promote the program, including local leaders, mobile phone messages, and posters at high-traffic areas. We did not examine the quality of ANC nor whether attending 4 or more ANC visits led to improved outcomes. However, assuming that more ANC visits are beneficial (5), promotion of the conditional cash transfer program would likely increase ANC attendance and improve maternal and neonatal health in the long term.

Our results suggest that women and their husbands who were unskilled, day or contracted laborers, women from the lowest wealth quintile, less educated , whose height was below 145cm, who identified as Vaishya, Shudra, and Muslim/other, should be prioritized with targeted methods (e.g. women's supporting groups, community leaders), transportation vouchers, and fee waivers. Additionally, pregnant women less than 18 years or greater than 35 years old, mothers of preterm babies, and multiparous women should be provided with flexible appointment hours, privacy-sensitive counseling, and re-booking options at postnatal discharge to ensure earlier engagement in subsequent pregnancies. Educating pregnant women about morbidity signs and symptoms during pregnancy may increase ANC service utilization, and possibly reduce maternal and neonatal mortality."

5.methods needs clear description about study participants like source population , sample size, tools, validity, reliability, eligibility criteria.

Thank you for the suggestion. We have revised our methods accordingly (page 6):

"**Study design**

This study is an observational secondary analysis of data collected during the Nepal Oil Massage Study (NOMS). The Nepal Oil Massage Study was a cluster-randomized community-based controlled trial that included mothers and their live and stillborn infants in 34 Village Development Committees (VDCs) in Sarlahi District, rural southern Nepal, from 2010 to 2017 (6). A total of 340 geographic clusters were randomized to receive either sunflower seed oil to massage for newborn babies or the standard mustard seed oil (6). Specifically, the NOMS treatment (i.e. either sunflower seed oil or the standard mustard seed oil for newborn babies' messages) was only implied after the birth of the baby, rather than during the pregnancy (6). Therefore, the treatment had no relevance to the number of ANC visits (6). Additional details about the NOMS study are available elsewhere, including the main NOMS publication (6).

Participants

The population for analysis consisted of married women of reproductive age for incident pregnancies that resulted in at least one live birth within NOMS (6). Since the trial enrolled and followed women over several years, some women contributed more than one pregnancy to the analysis. We excluded pregnancies with missing values or stillbirths or abortions, only including live births for analysis. We did not include pregnancies resulting in fetal loss because the number of ANC visits were not collected on them. All pregnancies that resulted in at least one live birth were included, regardless of their intervention assignment, as intervention for the NOMS trial occurred after delivery, and our ANC outcome (i.e. completion of ⩾4 ANC visits) occurred before delivery.

Married women of childbearing age were visited by locally residing female workers for pregnancy surveillance visits every 5 weeks and enrolled with consent when pregnant (6). During monthly intervals from enrollment to pregnancy outcome, field workers conducted home visits to confirm pregnancy status, document morbidity symptoms in the prior 30 days, and measure weight, blood pressure, pulse, and body temperature (6)"

6.discussion missed comparison and implications of the findings

Thank you for the feedback. We have added more comparisons and implications of the findings in the discussion section (pages 11-13):

"Previous research has shown that higher education of husbands, with their increased involvement during their wives' pregnancies, could increase the likelihood of women attending ⩾4 ANC visits, thereby benefiting maternal health (14). Our study found that women whose husbands were employed in non-agricultural sectors, were more likely to attend ⩾4 ANC visits. This indicates the role husbands play in maternal health and antenatal care, independent of the wife's educational background.

We found a positive dose response between higher educational levels and the receipt of ⩾4 ANC visits. Prior studies using NDHS 2001, 2011, and 2016 data align with our findings, emphasizing the role of education in facilitating the use of maternal health services and the positive associations with higher number of ANC visits (8,13,22). Higher education enhances women's understanding of the importance of professional healthcare, broadening their accessibility and utilization (14,23). In Nepal, the adult literacy rate for women is 60%, lower than the 68% recorded for men (24). Literacy rates in NOMS were much lower than

for Nepal as a whole. Promoting education, particularly for girls, might increase ANC utilization. Health education campaigns targeted at illiterate women with financial incentives, may be short-term interventions that improve ANC attendances (22). Longer term interventions such as advocating for women's education and employment may increase ANC attendance and overall maternal and neonatal health in Nepal (25). In our study, women from wealthier households were more likely to have attended ⩾4 ANC visits, which is consistent with results from previous studies (14,19,22). Therefore, there is a need to tailor ANC services to better support women from poor and less educated backgrounds to promote more attendance of ANC care.

Our results suggested that the odds of ⩾4 ANC visits increased from 2010 through 2017, which likely reflects that more women knew about the conditional cash transfer program and made more use of it over this time period. Therefore, it is important to continue promoting the conditional cash transfer program through methods such as enhancing government financial support, improving community advocacy and engagement among marginalized women.

Pregnancy history and prior pregnancy outcomes

Our study found that first-time pregnant women were more likely to have ⩾4 ANC visits compared to those with parity ⩾1. Our finding is inconsistent with a prior study from Western Nepal, which found that primiparous women had lower probability of receiving the recommended number of ANC visits (18)."

7. Authors need to state the gap of the study under abstract and last paragraph of introduction.

Thank you for the suggestion. We have added the research gap in the abstract and introduction:

"However, few studies have investigated the factors linked to the completion of ⩾4 ANC visits in Nepal." (page 2)

"Multiple factors have been linked to ANC utilization in Nepal, including socioeconomic factors (e.g. education, wealth, caste, occupation), biological characteristics (e.g. ethnicity, age), and cash incentives to encourage the completion of ⩾4 visits (18,19). Although there have been national reports on antenatal care coverage and its components (2,4), few studies have examined multi-level factors associated with the completion of ⩾4 ANC visits among a large population in Nepal. ANC coverage remains uneven across Nepal and Sarlahi district is considered as a poor region of Nepal (2,4,6). Therefore, it is important to identify the district-specific factors associated with completing ⩾4 ANC visits, particularly for marginalized populations such as pregnant women in Sarlahi District, Nepal. Therefore, we aimed to assess the association between demographic factors, socioeconomic status (SES), pregnancy history, morbidity symptoms in pregnancy, and the frequency of ANC visits by women in Sarlahi district of Nepal using NOMS data. These findings will inform interventions to increase ANC visits and improve maternal and neonatal health in Nepal and possibly similar LMICs." (page 5)

8. Keywords are too much. Try to write critical word only

Thank you for the suggestion. We have reduced the keywords (page 3):

"Maternal health; maternal and newborn healthcare; pregnancy; antenatal care

Reviewer: 2
Dr. Ilir Hoxha, Kolegjin Heimerer, Dartmouth College Geisel School of Medicine
Comments to the Author:
Overall…
This is a very well executed and presented study on predictors of ANC visits in Nepal. I have only a few minor comments which you will find below.

Specific comments

Abstract
Looks good intro, methods, outcome

Q1 The sentence "This study investigates factors associated with attending ≥ 4 ANC visits in Sarlahi district of southern Nepal" does not sound complete… factors predicting ANC visits?

Thank you for the suggestion. We have revised it (page 2):

"Objective: To investigate factors associated with attending ⩾4 ANC visits in Sarlahi district of southern Nepal."

Q2 I don't see how the conclusion part of claim is supported by results of the study… seem to broad… and not sure I I would agree with that linkage… but maybe authors can justify…

"These findings underscore the importance of continuing and promoting the government's program and increasing awareness among women." The other one is good "Moreover, understanding these factors can guide interventions aimed at encouraging ANC uptake in the most vulnerable groups, subsequently reducing maternal-related adverse outcomes in LMICs.

Thank you for the question. We have revised the conclusion in the abstract to be "These findings demonstrate the importance of socio-economic factors including education, caste, wealth and occupation in completion of ≥4 ANC visits. In addition, biologic factors including birth spacing, pregnancy complications, and nutrition are important. The association with awareness of the government's conditional cash transfer program is a motivation for a full evaluation of whether expanding that program might improve prenatal care." (page 2). We got this conclusion as awareness of the government's conditional cash transfer ANC program was associated with higher odds of the receipt of ≥4 ANC visits. In our result section we stated (page 10):

"Additionally, women who were aware of the government's conditional cash transfer program promoting ⩾4 ANC visits and facility-based delivery were more likely to attend ⩾4

ANC visits compared to those who were unaware of the program (adjusted OR = 2.26, 95% CI [2.01, 2.54]).”
Article strengths
Very well presented… and honest… overview of strengths and limitations…

Q3 One of bullets “Utilized a population-based pregnancy cohort dataset,” seems confusing. How come? Didn’t you have a secondary analysis of a cluster RCT? That’s not a cohort design to me!
Thank you for the question. We have revised it to be “Utilized a population-based pregnancy trial with large sample size, enabling prospective measurements of potential risk factors throughout pregnancies.”.

The study design was described in detail here (page 6)

“This study is an observational secondary analysis of data collected during the Nepal Oil Massage Study (NOMS). The Nepal Oil Massage Study was a cluster-randomized community-based controlled trial that included mothers and their live and stillborn infants in 34 Village Development Committees (VDCs) in Sarlahi District, rural southern Nepal, from 2010 to 2017 (6). A total of 340 geographic clusters were randomized to receive either sunflower seed oil to massage for newborn babies or the standard mustard seed oil (6). Specifically, the NOMS treatment (i.e. either sunflower seed oil or the standard mustard seed oil for newborn babies’ messages) was only applied after the birth of the baby, rather than during pregnancy (6). Therefore, the treatment and accounting for randomization to clusters is not relevance to the outcome of the number of ANC visits attended (6). Additional details about the NOMS study are available elsewhere, including the main NOMS publication (6). ”
Background
Seems ok to me

Methods
Q4 Participants’ paragraph seems thin… you may also refer to other publications if the participants have been described in previous publications…
Measures and predictors well described… really well done… I miss some rational why they were included…
We have revised the participants’ paragraph to include more details, as described in our response to Reviewer 1, Q5. As for rationale to include the measures and predictors, please see our response to Reviewer 1, Q7.

Q5 Stat analysis seem to account for within person correlation. What about within-cluster correlation? Since this is a cluster RCT, this should be taken into account somehow… you need that typical cluster effect estimation and integration in the analysis…
same as clustering, although this is a RCT cluster ….
Thank you for the question. There is no reason to account for within cluster correlation, typical cluster effect estimation and integration, stratification, or treatment arm in our study, as treatment was only implied after the birth of the baby, rather than during the pregnancy, therefore it has no relevance to ANC numbers. Mothers were blinded to the type of oil they received. We have added relevant information in the manuscript as shown below (page 6):

“**Study design**

This study is an observational secondary analysis of data collected during the Nepal Oil Massage Study (NOMS). The Nepal Oil Massage Study was a cluster-randomized community-based controlled trial that included mothers and their live and stillborn infants in 34 Village Development Committees (VDCs) in Sarlahi District, rural southern Nepal, from 2010 to 2017 (6). A total of 340 geographic clusters were randomized to receive either sunflower seed oil to massage for newborn babies or the standard mustard seed oil (6). Specifically, the NOMS treatment (i.e. either sunflower seed oil or the standard mustard seed oil for newborn babies’ messages) was only applied after the birth of the baby, rather than during pregnancy (6). Therefore, the treatment and accounting for randomization to clusters is not relevance to the outcome of number of ANC visits (6).

Participants

All pregnancies that resulted in at least one live birth were included, regardless of their intervention assignment, as intervention of the NOMS trial happened after delivery, and our ANC outcome (i.e. completion of ⩾4 ANC visits) occurred before delivery.”

Results
Seem well organized and presented…

Discussion
Q6 Seems a bit confusing… would suggest better organization… maybe start with summary of result… them strengths and limitations… they try to explain findings in context of other findings… then extend to implications and then conclusion… right now it’s a bit messy…
Thank you for the suggestion. We have revised our discussion section accordingly (pages 11-14). Details of revisions are replied in our response to Review 1, Q6.

Reviewer: 3
Prof. Zoe Kelson, University of Exeter
Comments to the Author:
This study investigates factors associated with attending ≥ 4 ANC visits in Sarlahi district of southern Nepal.

Reviewer comments:

Q1 "A secondary analysis was conducted on data from the Nepal Oil Massage Study (NOMS), a cluster-randomized, community-based longitudinal pregnancy cohort study encompassing 34 Village Development Committees"
Can the authors please further comment on whether the analysed cohort in this study can be considered to be representative?
Thank you for this feedback. Yes, this study can be considered to be representative. We have added explanations in the manuscript (page 15):

“In addition, this was a trial in which the entire population of this geographic area was surveyed for pregnancy over the study period. Houses with women of child bearing age were visited every 5 weeks, and any women who missed a period was tested for pregnancy

(6). With the exception of a very small number of women who refused participation, this was representative of all women who became pregnant during the time period in that geographic area (6). Also, this is an area very similar to rural populations in the plains of northern India, southern Nepal, western Bangladesh, and southern and central Pakistan (6). However, the external generalizability may be limited given that factors contributing to the receipt of ⩾4 ANC visits, particularly in urban areas or places where the health care system varies, might be different."

Q2 "All pregnancies resulting in a live birth (n=31,867) were included in the model." [Abstract]
and
"The Nepal Oil Massage Study (NOMS), was a cluster-randomized community-based controlled trial that included mothers and their live and stillborn infants in 34 Village Development Committees (VDCs) in Sarlahi District, rural southern Nepal, from 2010 to 2017 (14)."
and
"We excluded pregnancies resulting in fetal loss or records with missing values or stillbirths for pregnancy outcomes, only considering live births for analysis. All pregnancies that resulted in at least one live birth were included, regardless of their intervention assignment."
Can the authors please clarify why only live births were considered in the study?

Thank you for the question. Our primary analysis outcome was number of ANC visit. We did not collect the number of ANC visits for women with fetal loss in our NOMS study. Additionally, if we include women with fetal loss, this may bias the analysis, as pregnancies with fetal loss often have shorter gestation, so they are less likely to have 4+ ANC visits compared to women with live births. Although there may be different risk factors for women with fetal loss and with live birth, the number of fetal losses in our population is relatively low, around 2660 among 31867 pregnancies. For these reasons, we restricted the analysis to pregnancies resulting in live birth. We have added the information in the manuscript (page 6):

"The population for analysis consisted of married women of reproductive age for incident pregnancies that resulted in at least one live birth from NOMS (6). Since the trial enrolled and followed women over several years, some women contributed more than one pregnancy to the analysis. We excluded pregnancies with missing values or stillbirths or abortions, only including live births for analysis. We did not include pregnancies resulting in fetal loss because the number of ANC visits were not collected on them. All pregnancies that resulted in at least one live birth were included, regardless of their intervention assignment, as intervention of the NOMS trial occurred after delivery, and our ANC outcome (i.e. completion of ⩾4 ANC visits) occurred before delivery."

Q3 "A total of 340 geographic clusters were randomized to receive either sunflower seed oil to massage for newborn babies or the standard mustard seed oil (14). Married women of childbearing age were visited for pregnancy surveillance visits every 5 weeks and enrolled – with consent – when pregnant. "
Can the authors please adjust for treatment arm in the analysis?

Thank you for the question. No, there is no reason to adjust for treatment arm as treatment was only applied after the birth of the baby, rather than during the pregnancy, thus it has no relevance to ANC numbers. Mothers were blinded to the type of oil they received. Please see our detailed response to Reviewer 2, Question 5.

Q4 Furthermore, can original recruitment design, clustering, and stratification variables please be adjusted for in the analyses?

Thank you for the question. NOMS recruitment was to enroll anyone in the geographic areas who became pregnancy, therefore there is no need to adjust for recruitment in the analysis. Cluster and stratification are relevant to adjust for the outcome of the NOMS trial (i.e. neonatal mortality outcome), but in this case, the intervention happened after the birth, so adjusting is not relevant here. Please see our detailed response to Reviewer 2, Question 5.

Q5 "The number of ANC visits women attended during pregnancy was obtained from maternal recall by study staff during home visits as soon as possible after delivery. Our primary ANC visit outcome was binarized as four or more ANC visits, and less than 4 ANC visits (including no visits). "
The potential for recall bias has been acknowledged by the authors in the study limitations. Can a sensitivity analysis please be conducted to explore if the same factors are associated with 3 and 5 ANC visits?

Thank you for the suggestion. We conducted sensitivity analysis to investigate the associations between same factors with the receipt of ≥3, ≥5, and ≥8 ANC visits. Please see the results tables below (pages 31-37)

Supplementary Table 2. Longitudinal risk factors for ≥3 ANC visits

	**Crude Odds Ratio**	**95% CI**	**Adjusted Odds Ratio**	**95% CI**
Women occupation				
Farmer	Reference	Reference	Reference	Reference
Does not work	**1.33****	**1.20, 1.47**	0.94	0.84, 1.05
Unskilled/day labor/contracted laborer	**0.80***	**0.67, 0.95**	0.95	0.78, 1.16
Business/private/government service	**3.10****	**2.46, 3.91**	**1.48****	**1.16, 1.89**
Husband occupation				
Farmer	Reference	Reference	Reference	Reference
Does not work	**1.46****	**1.26, 1.70**	1.18	1.00, 1.39
Unskilled laborer/day labor or contracted laborer	**0.94***	**0.88, 0.99**	**1.07***	**1.00, 1.15**
Business/private service/government service	**1.49****	**1.40, 1.60**	**1.24****	**1.15, 1.33**
Maternal age (years)				
18-35	Reference	Reference	Reference	Reference
<18	0.98	0.92, 1.04	**0.79****	**0.73, 0.86**
>35	**0.63****	**0.54, 0.74**	**0.80***	**0.66, 0.97**
Maternal height (cm)				
<145	Reference	Reference	Reference	Reference
145-150	**1.23****	**1.14, 1.33**	**1.13****	**1.04, 1.23**

≥150	**1.38****	**1.29, 1.49**	**1.15****	**1.06, 1.24**
Caste/ethnicity				
Brahmin and Chhetri	Reference	Reference	Reference	Reference
Vaishya	**0.59****	**0.51, 0.68**	**0.75****	**0.64, 0.88**
Shudra	**0.41****	**0.35, 0.47**	**0.66****	**0.55, 0.78**
Muslim and others	**0.55****	**0.47, 0.65**	**0.76****	**0.64, 0.91**
Women education (years)				
No schooling	Reference	Reference	Reference	Reference
1-5	**1.64****	**1.51, 1.79**	**1.40****	**1.28, 1.54**
>5	**2.76****	**2.59, 2.93**	**1.93****	**1.80, 2.08**
Wealth quintile				
Poorest	Reference	Reference	Reference	Reference
Poorer	**1.40****	**1.31, 1.51**	**1.20****	**1.11, 1.30**
Middle	**1.67****	**1.55, 1.80**	**1.32****	**1.21, 1.43**
Richer	**1.96****	**1.82, 2.11**	**1.33****	**1.22, 1.46**
Richest	**2.49****	**2.30, 2.69**	**1.39****	**1.26, 1.53**
Gestational age (weeks)				
Term (≥37)	Reference	Reference	Reference	Reference
Mildly preterm (34-37)	**0.74****	**0.69, 0.80**	**0.79****	**0.73, 0.85**
Moderately preterm (<34)	**0.47****	**0.41, 0.52**	**0.51****	**0.44, 0.58**
Multiple birth				
Singleton	Reference	Reference	Reference	Reference
Twin/Triplet	0.86	0.66, 1.11	1.25	0.94, 1.66
Parity				
No Prior Pregnancy	Reference	Reference	Reference	Reference
Prior pregnancy but no live or stillbirth	**1.30****	**1.11, 1.53**	**0.52****	**0.37, 0.74**
Parity ≥1	**0.67****	**0.64, 0.71**	**0.63****	**0.59, 0.68**
Interpregnancy Interval (months)				
18-36	Reference	Reference	Reference	Reference
<18	1.00	0.95, 1.06	0.97	0.91, 1.04
>36	**1.27****	**1.17, 1.38**	**1.22****	**1.12, 1.34**
No prior pregnancy	**1.51****	**1.42, 1.61**	-	-
Prior live birth (LB) died				
Prior LB but not died	Reference	Reference	Reference	Reference
Prior LB died	**0.89****	**0.83, 0.97**	1.02	0.94, 1.11
Prior pregnancy but no LB	**2.06****	**1.80, 2.36**	**2.61****	**1.92, 3.55**
No prior pregnancy	**1.48****	**1.41, 1.56**	-	-
Prior pregnancy ended in stillbirth (SB)				
Prior pregnancy but no SB	Reference	Reference	Reference	Reference
Prior SB	**1.17****	**1.04, 1.31**	1.11	0.96, 1.29
No prior pregnancy	**1.47****	**1.40, 1.55**	-	-
Conditional cash transfer program knowledge	**2.57****	**2.36, 2.80**	**2.01****	**1.83, 2.21**
Year of childbirth	**1.20****	**1.18, 1.21**	**1.18****	**1.16, 1.20**
Fever				
No	Reference	Reference	Reference	Reference
Yes	0.98	0.91, 1.06	1.04	0.96, 1.13
Tachycardia				
No	Reference	Reference	Reference	Reference

Yes	1.01	0.97, 1.06	0.98	0.93, 1.03
Hypertension				
No	Reference	Reference	Reference	Reference
Yes	0.90	0.81, 1.00	0.92	0.82, 1.04
Respiratory problem				
No	Reference	Reference	Reference	Reference
Yes	**1.19****	**1.14, 1.25**	**1.14****	**1.08, 1.21**
Poor appetite, nausea & vomiting				
No	Reference	Reference	Reference	Reference
Yes	**1.29****	**1.23, 1.35**	**1.17****	**1.11, 1.23**
Vaginal bleeding				
No	Reference	Reference	Reference	Reference
Yes	**1.34****	**1.12, 1.60**	**1.31****	**1.07, 1.59**
Swelling of hands and face				
No	Reference	Reference	Reference	Reference
Yes	**1.30****	**1.17, 1.44**	**1.30****	**1.16, 1.45**
Sexually transmitted illness				
No	Reference	Reference	Reference	Reference
Yes	**1.22****	**1.15, 1.29**	**1.26****	**1.19, 1.34**

Note: ** p<0.01, * p<0.05

*Morbidity symptoms in pregnancy are for presence at any time in either the 2nd and/or 3rd trimesters

Supplementary Table 3. Longitudinal risk factors for ≥5 ANC visits

	**Crude Odds Ratio**	**95% CI**	**Adjusted Odds Ratio**	**95% CI**
Women occupation				
Farmer	Reference	Reference	Reference	Reference
Does not work	**1.26***	**1.03, 1.54**	0.84	0.68, 1.05
Unskilled/day labor/contracted laborer	**0.48****	**0.31, 0.75**	0.73	0.45, 1.18
Business/private/government service	**3.72****	**2.71, 5.10**	**1.48***	**1.05, 2.08**
Husband occupation				
Farmer	Reference	Reference	Reference	Reference
Does not work	**1.49****	**1.15, 1.93**	1.05	0.80, 1.38
Unskilled laborer/day labor or contracted laborer	**0.88***	**0.78, 0.98**	1.10	0.97, 1.25
Business/private service/government service	**1.89****	**1.68, 2.12**	**1.51****	**1.33, 1.70**
Maternal age (years)				
18-35	Reference	Reference	Reference	Reference
<18	1.00	0.88, 1.12	**0.81****	**0.71, 0.94**
>35	**0.61****	**0.43, 0.87**	0.95	0.66, 1.37
Maternal height (cm)				
<145	Reference	Reference	Reference	Reference
145-150	**1.32****	**1.13, 1.55**	1.17	0.99, 1.38

≥150	**1.67****	**1.44, 1.93**	**1.27****	**1.09, 1.49**
Caste/ethnicity				
Brahmin and Chhetri	Reference	Reference	Reference	Reference
Vaishya	**0.41****	**0.34, 0.49**	**0.59****	**0.48, 0.71**
Shudra	**0.24****	**0.19, 0.29**	**0.48****	**0.37, 0.62**
Muslim and others	**0.38****	**0.30, 0.47**	**0.63****	**0.49, 0.81**
Women education (years)				
No schooling	Reference	Reference	Reference	Reference
1-5	**1.79****	**1.54, 2.09**	**1.44****	**1.22, 1.70**
>5	**3.22****	**2.93, 3.53**	**1.92****	**1.71, 2.16**
Wealth quintile				
Poorest	Reference	Reference	Reference	Reference
Poorer	**1.40****	**1.18, 1.65**	1.13	0.95, 1.35
Middle	**1.60****	**1.36, 1.88**	1.13	0.94, 1.35
Richer	**2.35****	**2.02, 2.74**	**1.39****	**1.16, 1.66**
Richest	**3.28****	**2.83, 3.80**	**1.49****	**1.24, 1.79**
Gestational age (weeks)				
Term (≥37)	Reference	Reference	Reference	Reference
Mildly preterm (34-37)	**0.73****	**0.62, 0.84**	**0.79****	**0.67, 0.93**
Moderately preterm (<34)	**0.42****	**0.31, 0.56**	**0.49****	**0.35, 0.67**
Multiple birth				
Singleton	Reference	Reference	Reference	Reference
Twin/Triplet	1.36	0.90, 2.07	**1.94****	**1.21, 3.09**
Parity				
No Prior Pregnancy	Reference	Reference	Reference	Reference
Prior pregnancy but no live or stillbirth	**1.81****	**1.47, 2.23**	**0.58***	**0.35, 0.98**
Parity ≥1	**0.58****	**0.53, 0.64**	**0.60****	**0.52, 0.68**
Interpregnancy Interval (months)				
18-36	Reference	Reference	Reference	Reference
<18	1.05	0.93, 1.18	0.93	0.82, 1.06
>36	**1.49****	**1.28, 1.75**	**1.37****	**1.16, 1.61**
No prior pregnancy	**1.77****	**1.57, 1.99**	-	-
Prior live birth (LB) died				
Prior LB but not died	Reference	Reference	Reference	Reference
Prior LB died	0.99	0.84, 1.15	**1.19***	**1.01, 1.40**
Prior pregnancy but no LB	**3.24****	**2.71, 3.87**	**3.44****	**2.17, 5.45**
No prior pregnancy	**1.76****	**1.60, 1.93**	-	-
Prior pregnancy ended in stillbirth (SB)				
Prior pregnancy but no SB	Reference	Reference	Reference	Reference
Prior SB	**1.25***	**1.01, 1.54**	1.06	0.78, 1.46
No prior pregnancy	**1.64****	**1.50, 1.79**	-	-
Conditional cash transfer program knowledge	**2.34****	**1.91, 2.88**	**1.60****	**1.29, 2.00**
Year of childbirth	**1.24****	**1.21, 1.28**	**1.21****	**1.18, 1.25**
Fever				
No	Reference	Reference	Reference	Reference
Yes	0.88	0.76, 1.01	0.96	0.83, 1.12
Tachycardia				
No	Reference	Reference	Reference	Reference

Yes	0.98	0.90, 1.07	0.97	0.89, 1.07
Hypertension				
No	Reference	Reference	Reference	Reference
Yes	0.84	0.67, 1.04	0.83	0.66, 1.06
Respiratory problem				
No	Reference	Reference	Reference	Reference
Yes	**1.16****	**1.06, 1.27**	1.10	1.00, 1.21
Poor appetite, nausea & vomiting				
No	Reference	Reference	Reference	Reference
Yes	**1.26****	**1.16, 1.38**	**1.14****	**1.04, 1.26**
Vaginal bleeding				
No	Reference	Reference	Reference	Reference
Yes	**1.76****	**1.35, 2.29**	**1.80****	**1.36, 2.38**
Swelling of hands and face				
No	Reference	Reference	Reference	Reference
Yes	**1.44****	**1.21, 1.70**	**1.45****	**1.21, 1.74**
Sexually transmitted illness				
No	Reference	Reference	Reference	Reference
Yes	**1.25****	**1.13, 1.38**	**1.27****	**1.14, 1.41**

Note: ** p<0.01, * p<0.05

*Morbidity symptoms in pregnancy are for presence at any time in either the 2nd and/or 3rd trimesters

Supplementary Table 4. Longitudinal risk factors for ≥8 ANC visits

	**Crude Odds Ratio**	**95% CI**	**Adjusted Odds Ratio**	**95% CI**
Women occupation				
Farmer	Reference	Reference	Reference	Reference
Does not work	1.67	0.92, 3.05	1.44	0.73, 2.86
Unskilled/day labor/contracted laborer	0.39	0.09, 1.73	0.85	0.18, 4.03
Business/private/government service	**5.11****	**2.31, 11.32**	2.26	0.96, 5.36
Husband occupation				
Farmer	Reference	Reference	Reference	Reference
Does not work	1.66	0.90, 3.07	0.97	0.51, 1.85
Unskilled laborer/day labor or contracted laborer	0.87	0.65, 1.16	1.21	0.89, 1.64
Business/private service/government service	**1.94****	**1.46, 2.58**	**1.35***	**1.01, 1.80**
Maternal age (years)				
18-35	Reference	Reference	Reference	Reference
<18	0.77	0.55, 1.07	**0.62****	**0.43, 0.88**
>35	1.12	0.57, 2.18	1.95	0.92, 4.12
Maternal height (cm)				

<145	Reference	Reference	Reference	Reference
145-150	**1.69***	**1.07, 2.66**	1.50	0.93, 2.42
≥150	**2.52****	**1.65, 3.84**	**1.89****	**1.20, 2.96**
Caste/ethnicity				
Brahmin and Chhetri	Reference	Reference	Reference	Reference
Vaishya	**0.29****	**0.20, 0.42**	**0.42****	**0.28, 0.61**
Shudra	**0.14****	**0.09, 0.21**	**0.35****	**0.20, 0.61**
Muslim and others	**0.27****	**0.17, 0.45**	**0.47****	**0.27, 0.82**
Women education (years)				
No schooling	Reference	Reference	Reference	Reference
1-5	**1.96****	**1.31, 2.92**	**1.59***	**1.05, 2.42**
>5	**4.21****	**3.34, 5.32**	**2.35****	**1.74, 3.18**
Wealth quintile				
Poorest	Reference	Reference	Reference	Reference
Poorer	1.55	0.97, 2.48	1.22	0.74, 2.01
Middle	**1.73***	**1.09, 2.74**	1.17	0.71, 1.92
Richer	**3.06****	**2.00, 4.67**	**1.66***	**1.03, 2.67**
Richest	**4.70****	**3.14, 7.05**	**1.87***	**1.16, 3.03**
Gestational age (weeks)				
Term (≥37)	Reference	Reference	Reference	Reference
Mildly preterm (34-37)	0.67	0.45, 1.01	0.75	0.49, 1.15
Moderately preterm (<34)	0.77	0.42, 1.40	0.87	0.45, 1.71
Multiple birth				
Singleton	Reference	Reference	Reference	Reference
Twin/Triplet	1.91	0.79, 4.62	2.17	0.77, 6.10
Parity				
No Prior Pregnancy	Reference	Reference	Reference	Reference
Prior pregnancy but no live or stillbirth	**3.00****	**2.01, 4.47**	0.65	0.19, 2.28
Parity ≥1	**0.57****	**0.45, 0.72**	**0.55****	**0.39, 0.77**
Interpregnancy Interval (months)				
18-36	Reference	Reference	Reference	Reference
<18	1.21	0.89, 1.65	0.93	0.66, 1.30
>36	1.50	0.99, 2.25	1.22	0.79, 1.89
No prior pregnancy	**1.81****	**1.33, 2.45**	-	-
Prior live birth (LB) died				
Prior LB but not died	Reference	Reference	Reference	Reference
Prior LB died	1.36	0.93, 1.98	**1.78****	**1.20, 2.65**
Prior pregnancy but no LB	**5.81****	**4.06, 8.31**	**5.15****	**1.64, 16.22**
No prior pregnancy	**1.98****	**1.54, 2.55**	-	-
Prior pregnancy ended in stillbirth (SB)				
Prior pregnancy but no SB	Reference	Reference	Reference	Reference
Prior SB	1.39	0.83, 2.35	1.11	0.43, 2.87
No prior pregnancy	**1.58****	**1.26, 1.99**	-	-
Conditional cash transfer program knowledge	**2.54****	**1.44, 4.51**	1.78	0.94, 3.37
Year of childbirth	**1.29****	**1.20, 1.38**	**1.23****	**1.14, 1.33**
Fever				
No	Reference	Reference	Reference	Reference

Yes	0.67	0.44, 1.01	0.80	0.52, 1.23
Tachycardia				
No	Reference	Reference	Reference	Reference
Yes	0.90	0.72, 1.12	0.91	0.73, 1.15
Hypertension				
No	Reference	Reference	Reference	Reference
Yes	0.66	0.35, 1.24	0.61	0.31, 1.18
Respiratory problem				
No	Reference	Reference	Reference	Reference
Yes	1.17	0.94, 1.46	1.08	0.85, 1.37
Poor appetite, nausea & vomiting				
No	Reference	Reference	Reference	Reference
Yes	1.22	0.98, 1.51	1.09	0.86, 1.38
Vaginal bleeding				
No	Reference	Reference	Reference	Reference
Yes	**2.35****	**1.34, 4.11**	**2.17***	**1.19, 3.97**
Swelling of hands and face				
No	Reference	Reference	Reference	Reference
Yes	**1.78****	**1.21, 2.60**	**1.56***	**1.04, 2.34**
Sexually transmitted illness				
No	Reference	Reference	Reference	Reference
Yes	**1.29***	**1.01, 1.65**	1.28	0.98, 1.67

Note: ** p<0.01, * p<0.05

*Morbidity symptoms in pregnancy are for presence at any time in either the 2nd and/or 3rd trimesters

Q6 "Predictors including demographic, SES, and obstetric and medical history collected at enrollment. Pregnancy information such as morbidity symptoms were collected monthly throughout pregnancy, including morbidity recall and measurement of pulse, temperature and blood pressure. As soon as possible after birth, study staff interviewed the mother about labor and delivery and recorded the pregnancy outcome (e.g., live births, stillbirths, multiple births). Awareness of a government cash transfer program that incentivized women to receive ANC and deliver at a facility were noted through maternal recall. "
Can the authors please confirm if there was any missing covariate data?

Thank you for the question. We report the number and proportion of missing covariate data in Table 1 (“Characteristics of the full analytic sample, overall and stratified by ⩾4 ANC visits”) on pages 20-25 of the manuscript.

Q7 "We quantified the association between receipt/attendance of ≥ 4 ANC visits and socioeconomic, demographic, morbidity, and pregnancy history factors using logistic regression; Generalized Estimating Equations were used to account for multiple pregnancies per woman. " [Abstract]
and
"To examine factors associated with the completion of ≥ 4 ANC visits, we fit Generalized Estimating Equation (GEE) models with a logit link and an exchangeable working correlation structure, to account for the within-person correlation arising from including more than one pregnancy for many women in the study. We presented both the crude and adjusted odds

ratio (OR) of ≥ 4 ANC visits with the corresponding 95% confidence intervals (CIs)." Appropriate modelling methods have been applied.

Can the covariates included in the adjusted models please be specified in the Statistical analysis section too?

Thank you for the suggestion. We revised the Statistical Analysis section to specify all covariates included in the models (page 8):

"To examine factors associated with the completion of ⩾4 ANC visits, we fit Generalized Estimating Equation (GEE) models with a logit link and an exchangeable working correlation structure, to account for the within-person correlation arising from including more than one pregnancy for many women in the study. The predictors included women's occupation, husband's occupation, maternal age, maternal height, caste/ethnicity, women's education, wealth quintile, gestational age at birth, multiple births, parity, interpregnancy interval, prior livebirths that died, prior pregnancies that ended in stillbirth, conditional cash transfer program knowledge, year of childbirth, morbidity symptoms in pregnancy at any time in either the 2nd and/or 3rd trimesters, including fever, tachycardia, hypertension, respiratory problems, poor appetite, nausea & vomiting, vaginal bleeding, swelling of hands and face, sexually transmitted illness. The binary outcome is the receipt of ⩾4 ANC visits. Each predictor was tested within a univariable logistic regression models via GEE. In the multivariable GEE logistic regression model, all the predictors were included in the model. We presented both the crude and adjusted odds ratio (OR) of ⩾4 ANC visits with the corresponding 95% confidence intervals (CIs). "

Q8 "A tenth (11.0%, n = 3,517) of pregnancies were missing the reported number of ANC visits. In our study population, accounting for pregnancies with missing number of ANC visits, there were 28.0% with ≥ 4 ANC visits and 61.0% pregnancies with < 4 ANC visits, with the average number of ANC visits in the whole analyses sample being 2.4 ± 1.8."
Can the authors please clarify how this missing outcome data (i.e. missing number of ANC visits) has been accounted for in this assessment of frequencies?
Thank you for the question. To calculate the 11%, the numerator is the number of pregnancies with a missing ANC visit count, and the denominator is the total number of pregnancies (31,867), including both missing and non-missing ANC counts.

Q9 "Among 28,350 pregnancies with non-missing numbers of ANC visits"
Can the authors please confirm whether models have been developed using complete outcome data (i.e. 28,350 pregnancies, with reported ANC numbers)?

Thank you for the question. Yes. Only 28,350 pregnancies with non-missing numbers of ANC visits had ANC visits, and thus were included in the analysis model. We have added this information in the manuscript (page 9):

"Only 28,350 pregnancies with non-missing numbers of ANC visits were included in the analysis models."

Q10 Furthermore, can the authors please comment on whether missing number of reported ANCs be considered to be missing at random?

Thank you for the feedback. We do not know if they were missing at random. However, in comparing people with or without ANC number of visits, they appear to be similar when comparing the number of characteristics used as predictors in our models. We have added this information on page 9.

Q11 "From 2010 to 2017, women had an increased probability of attending ≥ 4 ANC visits per year (adjusted OR = 1.19, 95% CI [1.17, 1.22]). The prevalence of ≥ 4 ANC visits by child's birth year increased across time as follows: 64 (16.9%) in 2010, 510 (18.1%) in 2011, 714 (23.7%) in 2012, 576 (19.9%) in 2013, 1,836 (31.6%) in 2014, 2,328 (40.8%) in 2015, 2,689 (37.0%) in 2016, and 190 (43.1%) in 2017, not including missing values in the denominator"
and
"The prevalence of awareness of the government's conditional cash transfer program promoting ≥ 4 ANC visits increased over time: 254 (66.8%) in 2010, 2,286 (82.1%) in 2011, 2,472 (84.3%) in 2012, 2,470 (88.1%) in 2013, 5,408 (93.7%) in 2014, 5,400 (94.7%) in 2015, 6,891 (95.1%) in 2016, and 428 (96.8%) in 2017, not including missing values in the denominator. "
Can the authors please consider exploring changes over time statistically, in a formal trend analysis?
Thank you for the suggestion. We conducted formal trend analysis to investigate trends across child's birth years for both awareness of the government's conditional cash transfer program and 4+ ANC visits. We have added the results of trend analysis in the manuscript (page 10):

"We conducted a formal trend analysis to investigate trends across the child's birth years for both awareness of the government's conditional cash transfer program and 4+ ANC visits. There were increasing trends across the study period (2010-2017) for both awareness of the conditional cash transfer program (rising from 66.8% to 96.8%) and completion of ≥4 ANC visits (rising from 16.9% to 43.1%). These trends were statistically significant according to both Cuzick's non-parametric test for trend (Cuzick z=30.02 and 26.94, respectively; both p<0.001) and Kendall's tau correlation coefficients (Kendall's tau-b=0.15 and 0.14, respectively; both p <0.001), indicating an improvement in both metrics over time."

Q12 "Table 1. Characteristics of the whole analyses sample and stratified by ≥ 4 ANC visits or not "
and
"Table 2. Longitudinal risk factors for ≥ 4 ANC visits"
Can data on lifestyle factors, such as smoking or alcohol, please be considered for inclusion in the study?
Thank you for the question. We have added this information on page 7:

"We did not adjust for smoking or alcohol because the use of smoking or alcohol was low (tobacco: 1.1%, alcohol 0.3%) in the NOMS trial (5)."

"Challenges in establishing causal relationships due to the observational study design and potential confounding factors. • Maternal recall of ANC visits may introduce recall bias, with validity potentially differing. • Generalizability can be limited, particularly in urban settings

where factors influencing receipt of ≥4 ANC visits may differ. • Self-reported data introduces response biases, and the absence of first-trimester morbidity data and ANC quality evaluation may impact findings." [Strengths and Limitations of the Study]
and
"However, due to the limitations with observational studies, establishing a causal relationship between the factors discussed and the attendance of four or more ANC visits remains challenging. Also, we may have confounding such as unmeasured medical conditions during follow-up visits. We also asked the question about number of ANC visits with the exact wording used in the Nepal DHS. However, another study in this setting found that the validity of maternal recall of the number of ANC visits at six months postpartum is poor, although we do not know its validity when being asked at the time of delivery as in our study (24). In addition, the external generalizability may be low for this study given that factors contributing to the receipt of ≥ 4 ANC visits, particularly in urban areas, might be different. Recall bias is a potential limitation, as certain factors were ascertained through retrospective questioning. Most of our information is based on self-reported answers, introducing the possibility of response biases, including social desirability bias. Only the number of ANC visits was collected prospectively, and there was no evaluation of ANC quality. The presence of missing data for certain characteristics could introduce bias. Specifically, we did not have much morbidity data during the first trimester because it was difficult to identify and enroll women so early in pregnancy. Hence first trimester morbidity was excluded from the morbidity analysis."
A discussion on the study limitations is provided by the authors.

Thanks for providing a copy of the STROBE checklist.

Reviewer: 1
If you have selected 'Yes' above, please provide details of any competing interests.: Not applicable

Reviewer: 2
If you have selected 'Yes' above, please provide details of any competing interests.: Not applicable

Reviewer: 3
If you have selected 'Yes' above, please provide details of any competing interests.: Not applicable

We sincerely appreciate the reviewers' feedback on our manuscript. We believe that these comments have significantly strengthened the quality and clarity of our work. Thank you for continuing to consider our manuscript for publication in *BMJ Open*. We hope that our revisions have addressed all comments and suggestions. Should any additional changes be needed, we would be glad to make further modifications as requested. We look forward to hearing from you.

VERSION 2 - REVIEW

**Reviewer**	**2**
**Name**	**Hoxha, Ilir**
**Affiliation**	**Kolegjin Heimerer**
**Date**	**19-Jan-2026**
**COI**	

The authors have addressed all my comments...

**Reviewer**	**3**
**Name**	**Kelson, Zoe**
**Affiliation**	**University of Exeter, Mathematics**
**Date**	**04-Feb-2026**
**COI**	

Many thanks to the authors for their responses, clarifications and revisions.

Author response:

"We report the number and proportion of missing covariate data in Table 1 (“Characteristics of the full analytic sample, overall and stratified by ≥4 ANC visits”) on pages 20-25 of the manuscript."

Reviewer request:

Can the authors please assess whether missing data can be considered to be missing at random?

VERSION 2 - AUTHOR RESPONSE

Reviewer: 2
Dr. Ilir Hoxha, Kolegjin Heimerer, Dartmouth College Geisel School of Medicine
Comments to the Author:
The authors have addressed all my comments...
Thank you.

Reviewer: 3
Prof. Zoe Kelson, University of Exeter
Comments to the Author:
Many thanks to the authors for their responses, clarifications and revisions.
Thank you.

Author response:
"We report the number and proportion of missing covariate data in Table 1 (“Characteristics of the full analytic sample, overall and stratified by ≥4 ANC visits”) on pages 20-25 of the manuscript."
Reviewer request:
Can the authors please assess whether missing data can be considered to be missing at random?

Thank you for this question. We don’t know if data are missing at random but there do not appear to be differences between characteristics of those with and without missing data. We have added this sentence to the methods to address this question (page 6):

“We cannot say for sure that missing data are missing at random. However, there do not appear to be differences in characteristics available to us between missing and available data, implying missing at random is likely.”

Reviewer: 2
If you have selected ‘Yes’ above, please provide details of any competing interests.: No applicable...

Reviewer: 3
If you have selected ‘Yes’ above, please provide details of any competing interests.: Not applicable
